# Curcumin overcome primary gefitinib resistance in non-small-cell lung cancer cells through inducing autophagy-related cell death

**DOI:** 10.1186/s13046-019-1234-8

**Published:** 2019-06-13

**Authors:** Ping Chen, Han-Peng Huang, Yi Wang, Jun Jin, Wei-Guo Long, Kan Chen, Xiao-Hui Zhao, Chen-Guo Chen, Jian Li

**Affiliations:** 1 Department of Pulmonary Medicine, Affiliated Hospital of Jiangsu University, Zhenjing, 212001 China; 2 Center of Medical Experimental, Affiliated Hospital of Jiangsu University, Zhenjing, 212001 China; 3 Department of Pathology, Affiliated Hospital of Jiangsu University, Zhenjing, 212001 China

**Keywords:** Non-small-cell lung cancer, Epidermal growth factor receptor (EGFR), Tyrosine kinase inhibitor (TKI), Gefitinib, Curcumin, Resistance

## Abstract

**Background:**

Epidermal growth factor receptor (EGFR) tyrosine kinase inhibitors (TKIs) are being wildly used as target therapy in non-small-cell lung cancer (NSCLC). However, NSCLC patients with wild-type EGFR and KRAS mutation are primary resistant to EGFR-TKIs such as gefitinib. Curcumin has been known as a potential therapeutic agent for several major human cancers. In this study, we investigated the effect of curcumin on the reversal of gefitinib resistance in NSCLC cells as well as their molecular bases.

**Methods:**

H157 (wild-type EGFR and KARS mutation) and H1299 (wild-type EGFR and HRAS mutation) cells were treated with gefitinib or curcumin alone, or the two combination, and then cell viability, EGFR activity, expressions of Sp1 and Sp1-dependent proteins and receptor tyrosine kinases, markers of autophagy and apoptosis were examined by using CCK-8, colony formation, immunoblot, quantitative PCR, immunofluoscence, and flow cytometry assays. Also xenograft experiments were conduced to test the synergism of curcumin to gefitinib.

**Results:**

Our results showed that curcumin significantly enhanced inhibitory effect of gefitinib on primary gefitinib-resistant NSCLC cell lines H157 and H1299. Combination treatment with curcumin and gefitinib markedly downregulated EGFR activity through suppressing Sp1 and blocking interaction of Sp1 and HADC1, and markedly suppressed receptor tyrosine kinases as well as ERK/MEK and AKT/S6K pathways in the resistant NSCLC cells. Meanwhile, combination treatment of curcumin and gefitinib caused dramatic autophagy induction, autophagic cell death and autophagy-mediated apoptosis, compared to curcumin or gefitinib treatment alone, as evidenced by the findings that curcumin and gefitinib combination treatment-produced synergistic growth inhibition and apoptosis activation can be reversed by pharmacological autophagy inhibitors (Baf A1 or 3-MA) or knockdown of Beclin-1 or ATG7, also can be partially returned by pan-caspase inhibitor (Z-VAD-FMK) in H157 and H1299 cells. Xenograft experiments in vivo yielded similar results.

**Conclusions:**

These data indicate that the synergism of curcumin on gefitinib was autophagy dependent. Curcumin can be used as a sensitizer to enhance the efficacy of EGFR-TKIs and overcome the EGFR-TKI resistance in NSCLC patients with wild-type EGFR and/or KRAS mutation.

**Electronic supplementary material:**

The online version of this article (10.1186/s13046-019-1234-8) contains supplementary material, which is available to authorized users.

## Background

Epidermal growth factor receptor (EGFR), an oncogenic receptor tyrosine kinase, plays a key role in the development and progression of non-small-cell lung cancer (NSCLC) [1]. First-generation EGFR-tyrosine kinase inhibitors (TKIs) such as gefitinib and erlotinib reversibly bind to the ATP cleft within the EGFR kinase domain to block autophosphorylation of EGFR [2]. Dramatic tumor response and favorable clinical outcomes led to their widespread use in the first-line setting for patients with advanced NSCLC harboring activating EGFR mutation (a deletion in exon 19 or the L858R mutation in exon 21) [3, 4]. However, NSCLC patients initially response to these EGFR-TKIs almost invariably develop drug resistance [4, 5], which commonly arise through the acquisition of a second-site mutation (T790 M) within EGFR, or via activation of compensatory signaling pathways that bypass receptor and restore downstream oncogenic signaling [6, 7]. In addition, some NSCLC patients are intrinsically resistant to EGFR-TKIs, even though their tumors harbor activating mutation of EGFR [8]. Moreover, NSCLC overexpressing wild- type EGFR are often primary resistant, especially in tumor with concurrent KRAS mutation [9, 10]. However, the reasons for the innate resistance to EGFR-TKIs in wild-type EGFR and KRAS mutation NSCLC tumors still remain ill-defined. Therefore, the underlying mechanism of primary EGFR-TKI resistance need to be investigated in-depth. And developments of new EGFR-TKI sensitizers and combination strategies to surmount primary resistance to EGFR-TKIs are urgently required in patients with wild-type EGFR and KRAS mutation.

Several studies have showed that EGFR signaling regulates autophagy, a process involving sequestration and degradation of intracellular organelles and proteins in lysosomes, which function in cellular homeostasis and protection against a variety of disease including cancer [11]. The downstream targets of EGFR — PI3K, AKT, and mammalian target of rapamycin (mTOR) — are well established negative regulators of autophagy [12]. Furthermore, EGFR-TKIs induce autophagy in NSCLC [13–15] and other cancer cells [16]. However, the relation between EGFR-TKI resistance and autophagy is poorly understood. EGFR-TKI-induced autophagy in NSCLC cells has been postulated to exert either cytoprotective [14] or cytotoxic [15] effects.

Curcumin, a compound found in the plant *Curcuma longa*, has been demonstrated to inhibit the growth of various cancers [17] and sensitize tumor cells to chemotherapeutic drugs and ionizing radiation [18, 19]. We once reported that curcumin reversed cisplatin resistance in cisplatin-resistant lung cancer cells by suppressing the FA/BRCA pathway, a DNA cross-link damage repair pathway which regulates cellular sensitivity to DNA cross-linking agents [20]. Recently, several studies showed that curcumin is capable of inducing autophagy in different type of human cancer cells, including colon cancer, oral cancer, glioblastoma, uterine leiomyosarcoma and lung adenocarcinoma [21–25], suggesting that autophagy could play a certain role in the anticancer process of curcumin [26, 27]. A recent study reported that curcumin potentiated antitumor activity of gefitihib in gefitinib-resistant cells and xenograft mice model of NSCLC through inhibition of EGFR phosphorylation, and induction of EGFR degradation and apoptosis [28]. However, the underlying mechanism by curcumin sensitizing the NSCLC cells to gefitinib was not clearly elucidated. Since both EGFR-TKIs and curcumin induce autophagy in cancer cells, we infer that curcumin can enhance inhibitory effect of gefitinib on primary EGFR-TKI resistant NSCLC cells through augmenting gefitinib-induced autophagy and inducing autophagy-related cell death. To demonstrate the inference, we tested the synergism of curcumin on gefitinib in primary resistant NSCLC cells and examined whether the synergism of curcumin in combination with gefitinib is associated with autophagy induction and autophagy-mediated cell death, further investigate the relevant molecular bases involved in reversing of gefitinib resistance by curcumin.

## Methods

### Cell cultures

Three human NSCLC cell lines H157 (wild-type EGFR and KRAS mutation), H1299 (wild-type EGFR and NRAS mutation) and PC9 (EGFR exon 19 deletion) were obtained from American Type Culture Collection (Manassas, VA). Cells were maintained in 10% fetal bovine serum-supplemented RPMI 1640 medium (Sigma-Aldrich, St Louis, MO) and 1% penicillin-streptomycin (Invitrogen, Carlsbad, CA) at 37 °C in a humidified incubator containing 5% CO_2_. All cells were passaged for 3 months or less before the renewal from frozen, early-passage stocks obtained from the indicated sources. Cells were regularly tested for mycoplasma with the use of a MycoAlert Mycoplasma Detection Kit (Lonza, Switzerland).

### Cell viability assay

Cell viability was conducted by cell counting kit-8 (CCK-8, Dojndo, Japan) assays before and after treatment with indicated drugs according to the manufacturer^’^s instructions as previously described [20, 29]. For combination treatment of curcumin and gefitinib, CCK-8 assay data were converted to fraction of growth affected by the individual drug or the combination treated cells compared with untreated cells and analyzed using CalcuSyn Version 2.1 software (Biosoft, Gambride, UK) to determine whether the combination was synergistic. This program is based upon the Chou-Talalay equation [30], which calculates a combination index (CI). The combined effects of the two compounds can be summarized as follows: CI values <1, = 1 and >1 indicate synergistic, additive and antagonistic effects, respectively.

### Colony formation assay

Colony formation assay was performed as described previously [31]. Briefly, a density of 1000 cells per well seed onto 6-well culture plate and the indicated drugs or vehicle in DME containing 10% FBS was added to the culture at 3 day after seeding. The culture was continuously maintained for another 14 days and subjected to the colony formation assay. Colonies were visualized and countered under a microscope.

### Immunoblot assay

Cells were treated with the indicated drugs at various concentration, and proteins from whole cell lysates were prepared and determined using immunoblotting as previously described [31]. The antibodies used to determine the proteins in this study were described in Additional file 1: Supplementary materials and methods.

### RNA interference

Small interfering RNA (siRNA) siSp1, siSp3, siBeclin-1 and siATG7 and nonsilencing control siRNA were purchased from Qiagen (Duesseldorf, Germany). These siRNAs were transfected into cells with transfectamine 2000 Transfection Reagents in accordance with manufacturer’s instructions. After 48 h, the cells were harvested and subjected to immunoblot or RT-PCRassay, as previously described [29].

### Real-time quantitative PCR and PT-PCR

For real-time quantitative PCR, total RNA was extracted from various cell samples using Trizol reagent (Invitrogen, Carlsbad, CA). Reverse transcription were conducted using the Applied Biosystem^’^s Power SYBR Green PCR Master Mix and the reactions were run on an ABI 7500 Fast Real-time PCR system, as previously described [31]. For RT-PCR, products were resolved by electrophoresis on 1% agarose gels and visualized by ethidium bromide staining. The specific primary sequences of the genes detected in this study are shown in Additional file 2: Table S1.

### Immunofluorescence analysis

LC3-II puncta detection was performed as previously described [31]. Briefly, cells were seeded on coverslips, and treated with the indicated drugs before being fixed with 2% paraformaldehyde in PBS for 20 min at 37 °C. Following permeabilization for 3 min with PBS-0.2% Triton X-100, the cells were rinsed three times in PBS and incubated overnight at 4 °C with anti-LC3 antibody diluted 1:100. After three times washes in PBS, the cells were incubated for 1 h at room temperature with Alexa Fluor 488-conjugated secondary antibody at a 1:400 dilution. Then cells were counterstained with Hoechst 33342 for 5 min for nuclear staining, and mounted on glass slides. Cells were visualized using a Leica Confocol Laser Scanhing Microscope (Leica, Wetzlar, Germany).

### Sub-G1 population assay

Sub-G1 population was analyzed by flow cytometry as previously described [32]. Briefly, cells were treated with the indicated drugs for 24 h, harvested and fixed using 7% ethanol. And then cells were stained with mixture of 50 μg/mL propidium iodide, 0.2% Triton X-100, and 100 μg/mL RNAase, and performed using a FACS flow cytometer equipped with Modfit LT for Mac V 2.0 software (BD Biosciences, NJ).

### In vivo studies

Six-week-old female athymic nude BALBL/c mice were obtained from Charles River Laboratories (Wilmington, MA). All mice were handled in accordance with the protocols approved by the Institutional Animal Care and Use Committee of the Jiangsu University. Before starting the experiments, mice were maintained for 5 days in the temperature and humidity controlled condition. H157 and H1299 cells (6 × 10^6^ cells per mouse) were subcutaneously grafted into the right flanks. When tumor volume reached approximately 70 mm ± 10 mm^3^, mice were randomly allocated into groups of 6 animals to receive either vehicle control, gefitinib (100 mg/kg, one daily) alone, curcumin (1 g/kg, once daily) alone, or gefitinib and curcumin together. Doses of gefitinib and curcumin used in the mice with tumor were according to Lee et al. and Xu et al. experience [28, 33]. Gefitinib was suspended in saline and curcumin was prepared properly in propylene glycol and administered orally by gavage. Tumor volume was measured every 5 days using calipers. The average tumor volume in each group was expressed in mm^3^ and calculated according to the equation (lenth **×** width^2^)/2. At study termination, tumor xenografts were harvested and weighted. Tumor samples obtained were snap-frozen in liquid nitrogen and stored in − 80 °C for immunoblot analysis.

### Statistical analysis

Each experiment was performed at least three times, and all data are presented as the mean ± SD. Statistical analysis was performed by the unpaired 2-tailed Student *t* test or the Mann-Whitney U-test using the SPSS version 22.0 (SPSS Inc.; Chicago, IL). *P*-values <0.05 were considered significant (*, *P*<0.05; **, *P*<0.010; ***, *P*<0.001).

A complete description of the methods, including drugs and antibodies used in this study, cell proliferation assay, BrdUred incorporation assay, luciferase assay, plasmid transient transfection, caspase activity assay and immunofluorescence analysis are available in Additional file 1: Supplementary materials and methods.

## Results

### Curcumin enhances the inhibitory effect of gefitinib on gefitinib-resistant NSCLC cells by suppressing EGFR activity

**Fig. 1:**
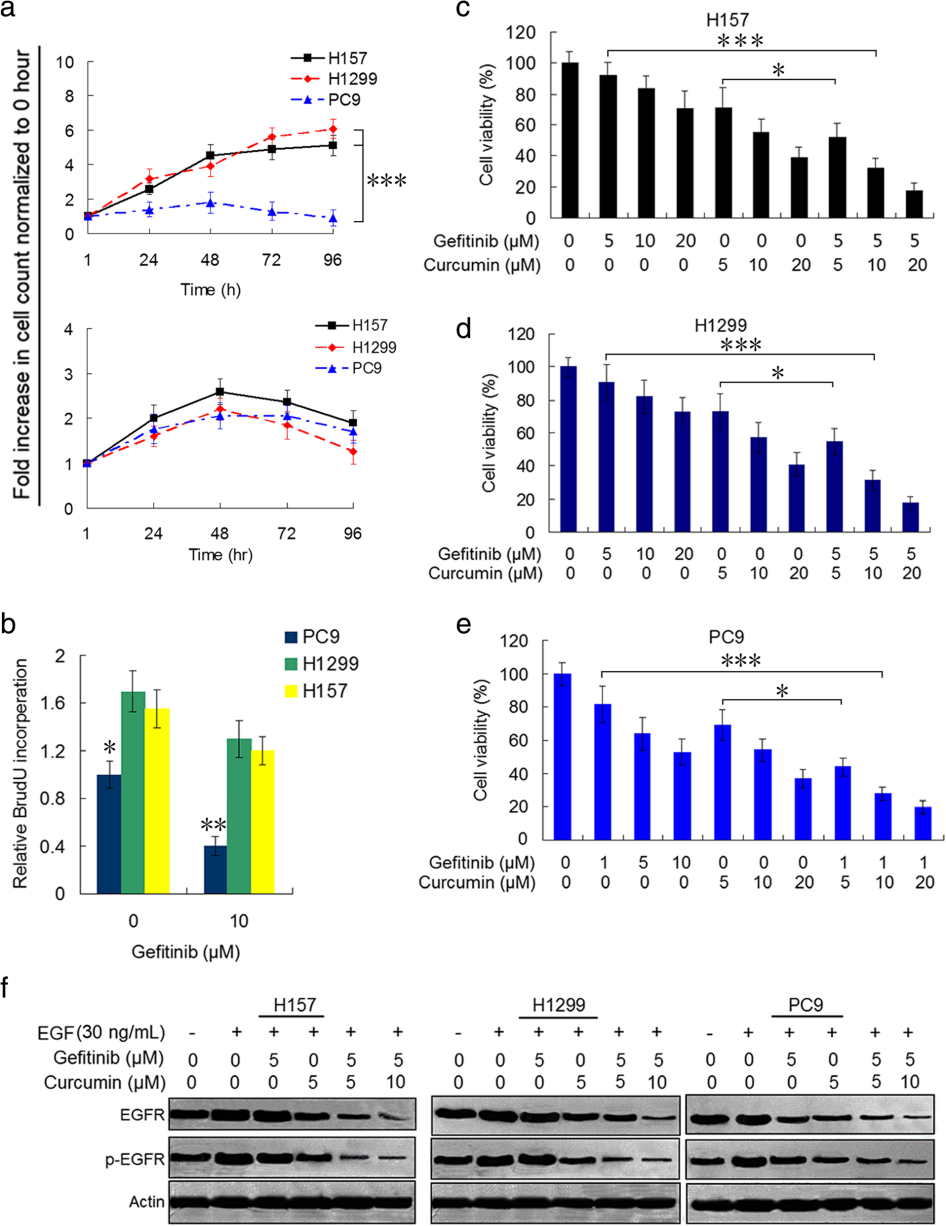
Curcumin enhances anticancer effect of gefitinib on NSCLC cell and suppresses EGFR activity. **a** H157, H1299 and PC9 cell lines were growth in complete media in the presence of 5 μM gefitinib (top), or 5 μM curcumin (nether) for 24, 48, 72, 96 h. Fold increase in cell counts normalized to zero hour counts of respective cell lines are represent (***P<0.001). **b** The three cell lines were grown in the presence DMSO or 10 μM gefitinib in complete media. BrdU substrate was added 48 h after drug treatment and assayed after 24 h. H157 **c**, H1299 **d** and PC9 **e** cells were treated with gefitinib, or curcumin alone, or the two combination at indicated concentrations for 48 h. Cell viability was measured by CCK-8 assay (*P<0.05; ***P<0.001). **f** H157, H1299 and PC9 cell lines were pre-treated with curcumin or gefitinib alone, or the two combination at indicated concentrations for 12 h, and then EGF (30 ng/mL) was added for 1 h. Immunoblot analysis was used to determine p-EGFR and total EGFR expression. Actin was used as aloading control in immunoblots. Similar results were obtained from three independent experiments. Typical immunoblots were presented in the Figure

Since NSCLCs with wild-type EGFR and KRAS mutation were shown to be primary resistant to EGFR-TKIs [9, 10], we examined the inhibitory effect of gefitinib on proliferation of NSCLC cells with different gene background. Resistance to gefitinib was reflected in H157 and H1299 total cell counts, recorded over time with 5 μM gefitinib treatment and expressed as fold increase over time compared to baseline (0 h) (Fig. 1a, upper). Conversely, treatment with the same concentration of gefitinib, PC9 cell growth was significantly reduced. Upon treatment with 5 μM curcumin, H157, H1299 and PC9 cell lines showed a similar proliferative inhibition (Fig. 1a, lower). Consistent with elevated proliferation rate, H157 and H1299 cells exhibited greater BrdU incorporation compared to PC9 cells, both in the absence and presence of gefitinib (Fig. 1b). Then we evaluated the ability of combination treatment with gefitinib and curcumin to inhibit the survival of the three NSCLC cell lines. Cells were treated with increasing concentrations of gefitinib and/or curcumin for 48 h, and survival inhibition was measured by CCK-8 assay. Compared with gefitinib or curcumin alone, all cells treated with combination of gefitinib and curcumin displayed significantly decreased viability (Fig. 1c-e). The CI values were all <1 (Additional file 3: Figure S1a-c), indicating that these was a synergistically inhibitory effect on the viability of the three NSCLC cell lines in all used combination concentrations. Clonogenic assay demonstrated that combination of gefitinib and curcumin markedly suppressed colony formation in H157, H1299 and PC9 cells compared to either gefitinib or curcumin treatment alone (Additional file 3: Figure S1e). However, the CI values of gefitinib plus curcumin at different combinations in PC9 cells were all close to 1 (Additional file 3: Figure S1c), which was much higher than those in gefitinib-resistant NSCLC cell lines H157 and H1299 (Additional file 3: Figure S1a and b), suggesting that the degree of gefitinib sensitization caused by curcumin is more pronounced in gefitinib-resistant cells than in gefitinib-sensitive cells.

We further examined that the effect of gefitinib and curcumin on EGFR activity in these NSCLC cells. The cells pre-treated with gefitinib, or curcumin alone, or the two drug combination for 12 h, were stimulated with EGF (30 ng) for 1 h. Pre-treatment with gefitinib alone barely affect EGFR activity induced by EGF in H157 and H1299 cell lines upon EGF exposure. While curcumin alone moderately reduced EGF-induced EGFR phosphorylation, combined curcumin with gefitinib markedly decreased phosphorylated and endogenous EGFR levels induced by EGF in two gefitinib-resistant cell lines (Fig. 1f and Additional file 3: Figure S2). In PC9 cells, however, EGF-induced phosphorylated and total EGFR levels were clearly reduced by pre-treatment with gefitinib or curcumin alone. In addition, the proteasome inhibitor, MG132 partially restored curcumin and gefitinib combination-induced EGFR downregulation in H157 cells (Additional file 3: Figure S3a). These results indicate that primary resistance to gefitinib in the NSCLC cells with wild-type EGFR and KRAS mutation is associated with insensitive to gefitinib-induced EGFR downregulation, and gefitinib and curcumin combination-induced EGFR downregulation partially through proteasome-induced degradation of EGFR protein.

### Combination of curcumin and gefitinib inhibits EGFR through suppressing Sp1 and HDAC1 binding induced EGFR transcription activity

**Fig. 2:**
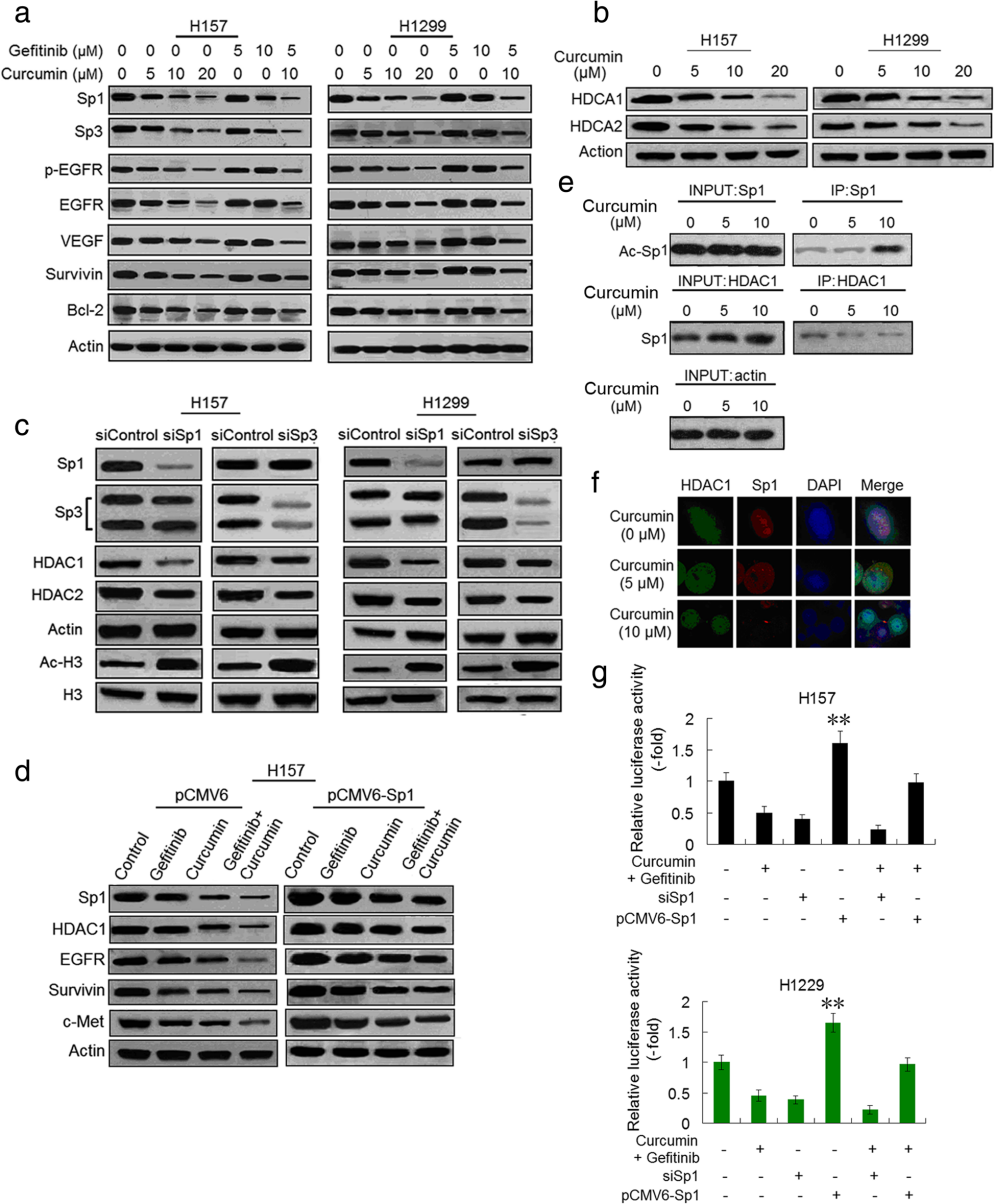
Combination of curcumin and gefitinib downregulates EGFR through inhibiting SP1 and HDAC1 binding induced-EGFR transcription activity. **a** H157 and H1299 cells were treated with gefitinib, or curcumin alone, or the two combination at indicated concentrations for 48 and evaluated for the protein expression as indicated by immunoblot analysis. **b** Curcumin-induced suppression of HDCA1 and HDCA2 proteins in H157 and H1299 cells. Protein expression was determined as outlined above in A. **c** Expressions of Sp1 and other indicated proteins in control and Sp1 or Sp3 knocked down cells were determined by immunoblotting in H157 and H1299 cells. **d** H157 cells were transfected with Sp1 plasmids (PCMV6-Sp1) and control plasmid DNA (PCMV6). After 8 h, cells were treated with 5 μM gefitinib, or 10 μM curcumin alone, or two drug combination for an additional 48 h. And then the expressions of Sp1, HDAC1, EGFR, Survivin and c-Met proteins were determined by immunoblotting. Similar results were obtained from three independent experiments. Typical immunoblots were presented in the Figure. **e** H157 cells were pre-incubated with curcumin and nuclear extracts were prepared. The immunoprecipitation was performed using anti-Sp1 and anti-HDAC1 antibodies. The immunoprecipitated pellets were analyzed by immunoblotting with anti-acetyl-lysine and anti-Sp1 antibodies. **f** H157 cells treated with DMSO or curcumin at indicated concentrations were stained for HDAC1 (green) and Sp1 (red) using immunofluorescence assay. **g** Effect of Sp1 on EGFR promoter activity. Cells were treated with curcumin (10 μM) plus gefitinib (5 μM), or transfected with siSp1 or plasmids containing Sp1 (PCMV6-Sp1), and luciferase activity was determined as described in Materials and Methods. (**P<0.01 compared with gefitinib plus curcumin, or siSp1, or gefitinib plus curcumin in combination with pCMV6-Sp1, respectively)

EGFR expression is modulated by multiple factors, including Sp transcription factors [34–36]. Thus we examined the effect of curcumin on Sp factors expression. Although curcumin can decrease Sp1, Sp2, Sp3 and Sp4 expressions in H157 cells, only downregulations of Sp1 and Sp3 reduced EGFR protein levels (Additional file 3: Figure S3b). Gefitinib alone slightly or moderately depressed Sp1 and Sp3 expression, but combined with curcumin further decreased Sp1 and Sp3 protein levels, which was accompanied by a parallel decrease of phosphorylated and endogenous EGFR, and other Sp1-dependent proteins, namely VEGF, Survivin and Bcl-2 expressions (Fig. 2a and Additional file 3: Figure S4a). Sp1 and Sp3 regulate EGFR activity through interacting with HDAC1 and HDAC2 in cancer cells [37]. We demonstrated that curcumin inhibited HDAC1 and HDAC2 expressions in a dose-dependent manner (Fig. 2b and Additional file 3: Figure S4b). Then we investigated the function of Sp1 and Sp3 in regulating HDAC1 and HDAC2 in H157 and H1299 cells. Sp1 and Sp3 were knocked down with specific siRNAs (Additional file 3: Figure S1d). Sp1 and Sp3 silencing reduced HDAC1 and HDAC2 expressions and affect global histone acelylation levels (Fig. 2c and Additional file 3: Figure S4c). Moreover, treatment with mithramycin A (MIT), a reagent that blocks both Sp1 and Sp3 binding to GC boxes of HDAC1 and HDAC2 promoters [38], results in decrease of HDAC1 and HDAC2 which was accompanied by reduction of the receptor tyrosine kinases, such as EGFR, c-Met, Her-2, AXL and IGF1R (Additional file 3: Figure S3c). The findings indicate that both Sp1 and Sp2 are required for HDAC1 and HDAC2 activation. However, the decrease of HDAC1 expression mediated by Sp1 depletion was more significant (Fig. 2c and Additional file 3: Figure S4c). Reduction of HDAC2 expression by depleting Sp1 or Sp3 was relative minimal. To confirm the role of Sp1 and HDAC1 in EGFR expression following gefitinib and curcumin treatment, Sp1 ectopic overexpressed H157 cell line was established by plasmid transfection. Sp1 ectopic overexpression elevated levels of HDAC1 and EGFR, retarded gefitinib and curcumin combination-induced EGFR downregulation (Fig. 2d and Additional file 3: Figure S4d). To examine whether Sp1 was acetylated or bound to HDAC1 and what effect curcumin had on these interactions, cell nuclear lysates were prepared and then immunoprecipitated with anti-Sp1 or anti-HDAC1 antibodies followed by immunoblot analysis with antibodies against acetyl-lysine or Sp1. Results showed that one band located at − 90 KD could be identified by anti-acetyl-lysine antibody in anti-Sp1 immunoprecipitants in the curcumin treated group. After normalization for the amount of protein present, a decreased binding of HDAC1 to Sp1 was found in the presence of curcumin (Fig. 2e). The immunofluorescence assay also revealed a reduced co-location between Sp1 and HDAC1 in nuclei of cells treated with curcumin (Fig. 2f).

To further determine if the effect of curcumin and gefitinib combination on EGFR was mediated by affecting EGFR transcription activity, we performed luciferase assay. As shown in Fig. 2g, Sp1 silencing and overexpression enhanced and reversed curcumin and gefitinib combination-induced decrease of EGFR promotor activity respectively. Likewise, quantitative PCR analysis showed that knockdown and overexpression of Sp1 potentiated and returned curcumin plus gefitinib-induced EGFR mRNA expression downregulation in H157 and H1299 cells respectively (Additional file 3: Figure S3d). Collectively, these data indicated that the diminished interaction between Sp1 and HDAC1 caused by curcumin contributes the two drugs combination-induced EGFR activity downregulation.

### Curcumin and gefitinib synergistically inhibit the receptor tyrosin kinas signaling and ERK-AKT pathways

**Fig. 3:**
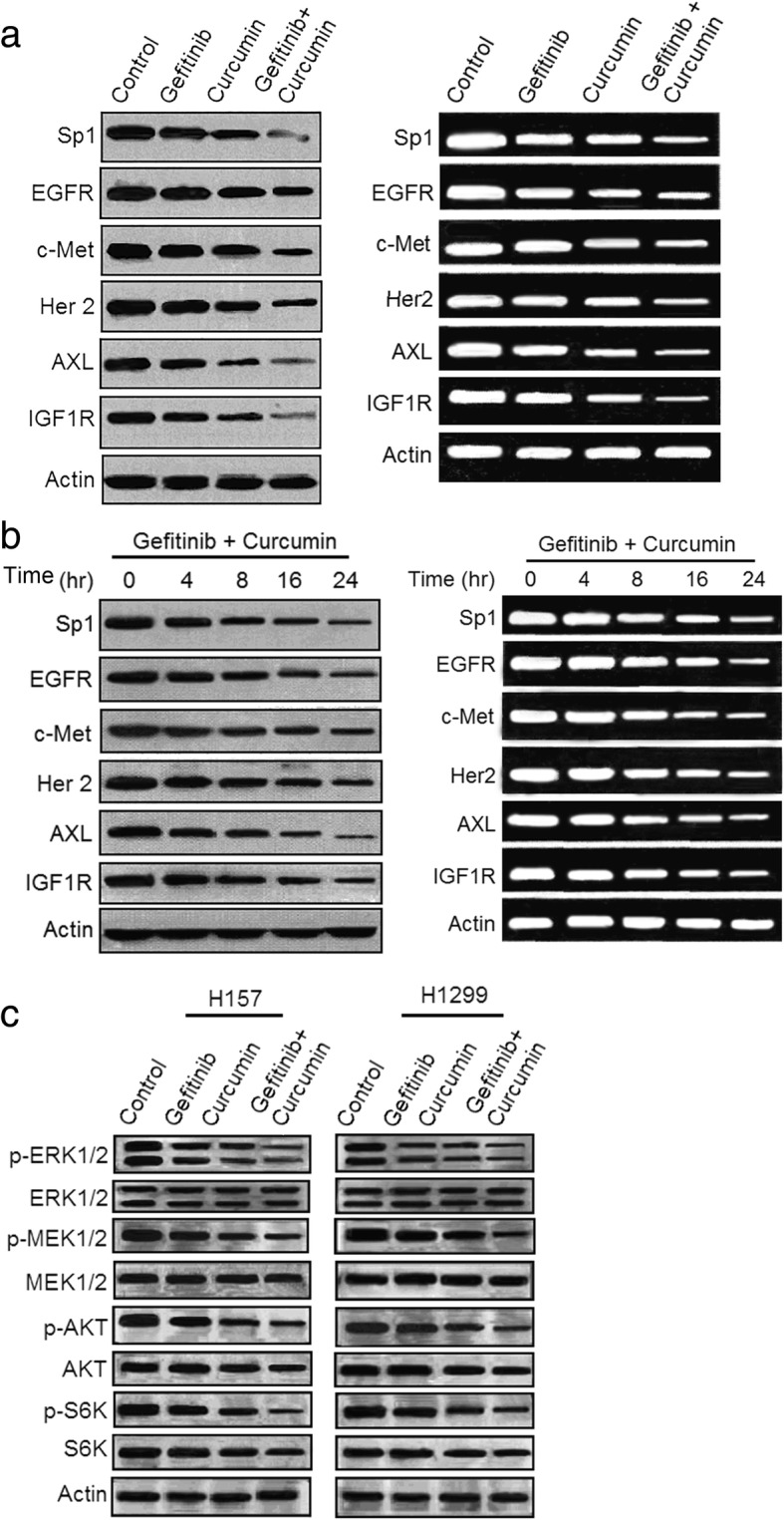
Combination of curcumin and gefitinib markedly depresses the receptor tyrosine kinase signaling pathways in gefiinib-resistant NSCLC cells. **a** H157 cells were treated with 5 μM gefitinib, or 5 μM curcumin alone, or the two combination for 48 h. Cell lysates and total mRNA were prepared and subjected to immunoblotting and RT-PCR analysis for detecting proteins and mRNA as indicated. **b** The combination of curcumin (5 μM) and gefitinib (5 μM) time-dependently suppressed expressions of proteins and mRNAs as indicated. **c** H157 and H1299 cells were treated with gefitinib (5 μM), or curcumin (5 μM) alone, or the two combination for 48 h. The whole-cell lysates were prepared and subjected to immuoblotting analysis for detecting phosphorylations of ERK1/2, MEK1/2, AKT and S6K proteins. Similar results were obtained from three independent experiments. Typical immunoblots were presented in the Figure

It is known that bypass receptor tyrosine kinase amplification and activation of compensatory signaling pathways are involved in the development of EGFR-TKI resistance in NSCLC [7, 39]. To deeply probe the molecular basis of gefitimb sensitization by curcumin in primary EGFR-TKI resistant NSCLC cells, we tested the protein and mRNA levels of reported receptor tyrosine kinase. Results showed that H157 cells treated with either gefitinib or curcumin alone displayed slight or moderately reduction of protein levels of EGFR, c-Met, Her-2, AXL and IGF1R, whereas combination treatment of gefitinib and curcumin showed apparent decrease of these protein expressions (Fig. 3a and Additional file 3: Figure S5a). Time-dependent downregulations of these receptor tyrosine kinases were observed in H157 cell treated with the two drug combination (Fig. 3b and Additional file 3: Figure S5b). To determine if the decreased expression of the receptor tyrosine kinases induced by the two drugs result from transcription inhibition, we also performed the PCR analysis. As show in Fig. 3a and b (right), in H157 cells, the inhibitory effect on the receptor tyrosine kinase induced by the two drug combination occurred at the transcriptional level, suggesting that combination of curcumin gefitinib remarkably suppress multiple receptor tyrosine kinase pathways in gefitinib-resistant NSCLC cells at both the transcriptional and protein levels.

EGFR regulates multiple signaling pathways via activation of downstream kinase, such as the ERK/MEK and AKT/S6K pathways. Figure 3c and Additional file 3: Figure S5c show the effect of the drugs on the ERK/MEK and AKT/S6K phosphorylation levels in H157 and H1299 cells. Gefitinib alone had a minor affect on phosphorylation of ERK1/2, MEK1/2, AKT and S6K, the two drug combination further reduced phosphorylation levels of all kinases detected in this study. The results indicate that the combination treatment blocks EGFR downstream tyrosine kinases that have key roles in the pro-oncogenic signaling of NSCLC, thereby potentiating the inhibitory effects of gefitinib on gefitinib-resistant NSCLC cells.

### Combined curcumin with gefitinib augments autophagy induction in NSCLC cells through suppressing Sp1/EGFR signaling

**Fig. 4:**
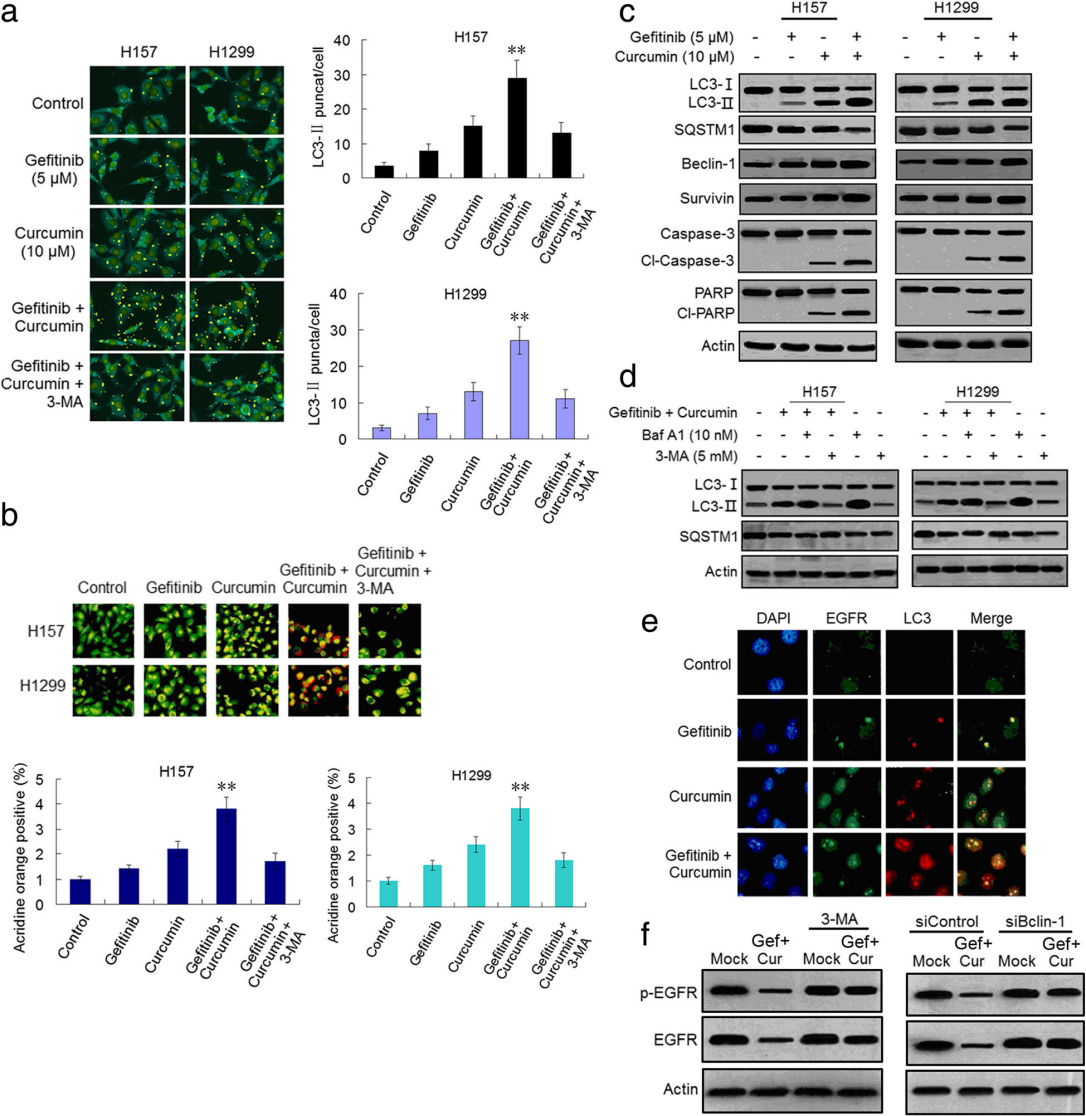
Combination of curcumin and gefitinib significantly potentiates induction of autophagy and apoptosis. **a** H157 and H1299 cells were treated with gefitinib, or curcumin alone, or the two combination at indicated concentration for 48 h. Then the LC3-II puncta formation was determined using immunofluorescence analysis and imaged by confocal microscopy (left). The number of LC3-II puncta/cell was quantified by Image-Pro Plus 5.1 software (right). (**P<0.01 compared with gefitinib, or curcumin alone, or the two combination plus 3-MA, respectively). **b** H157 and H1299 cells were stained with acridine orange after drug treatments (5 μM of gefitinib or 10 μM of curcumin alone, or the two combination for 48 h) and analyzed by confocal fluorescent microscopy. Development of AVOs (orange) was observed (left). Quantification of AVOs using FACSanalysis (right) (** *P*<0.01 compared with gefitinib, or curcumin alone, or the two combination plus 3-MA, respectively). **c** H157 and H1299 cells were treated with gefitinib, or curcumin alone, or gefitinib plus curcumin at indicated concentration for 48 h. The autophagy-related and apoptosis activation-related proteins were analyzed by immunoblotting. **d** H157 and H1299 cells were treated with gefitinib plus curcumin in the absence and presence of Baf A1 or 3-MA. The autophagy-related proteins were analyzed by immunoblotting. **e** H157 and H1299 cells were treated with gefitinib, or curcumin alone, or gefitinib plus curcumin at indicated concentration for 48 h and then were fixed with methanol, immunostained with anti-EGFR (green), anti-LC3 (red), and DAPI (blue), and analyzed by confocal microscopy to determine the intracellular locations of EGFR and LC3. **f** H157 cells were treated with 5 μM gefitinib (Gef) plus 10 μM curcumin (Cur) for 48 h in the presence or absence of 3-MA or Beclin-1 knokdown. The phosphorylated and total EGFR were detected by immunoblotting. Similar results were obtained from three independent experiments. Typical immunoblots were presented in the Figure

It has been reported that curcumin induces autophagy in several cancer cell lines [39–41]. EGFR-TKIs have been also shown to activate autophagy in NSCLC and other cancer cells [42–44]. We hypothesize that combination of curcumin and gefitinib can effectively inhibit growth in gefitinib-resistant NSCLC cells through the mechanism of autophagy induction. Indeed, immunofluorescence (IF) microscope showed that getitinib or curcumin treatment alone resulted in slight or moderate increase of LC3-II puncta in H157 and H1299 cells (Fig. 4a). However, the number of puncta were dramatically increased following combination treatment of the two drugs comparing to either drug treatment alone (Fig. 4a). Combination treatment also markedly increased acidic vesiculcr organelles (AVO) detected using acridine orange (AO) stained compared to treatment with either of the two drugs (Fig. 4b). Moreover, the increased LC3-II puncta and AVO by gefitinib plus curcumin in H157 and H1299 cells were depressed by co-treatment with 3-MA which is a selective PI3K inhibitor and impedes autophagosome formation [45]. Furthermore, the potentiation of autophagy induction by the combination treatment was evidenced by augmented accumulation of LC3-II and reduction of SQSTM1 (Fig. 4c and Additional file 3: Figure S6a), which are two important markers for autophagy activation. Combination treatment-induced accumulation of LC3-II was further increased by Baf A1 which is a selective inhibitor of vacuolar H^+^-ATPase (V-ATPase) that blocks the autophagic degradation via lysosome [46], and was decreased by 3-MA (Fig. 4d and Additional file 3: Figure S6b), indicating increased autophagic flux formation by the combination treatment. In parallel with autophagy induction, the combination treatment led to elevation of cleaved-caspase-3 and cleaved-PARP and reduction of survivin (Fig. 4c and Additional file 3: Figure S6a), suggesting that combined gefitinib with curcumin concurrently induce autophagy and apoptosis.

Since gefitinib and curcumin combination-induced EGFR downregulation was mediated by repressing Sp1, Sp1 is involved in induction of autophagy and apoptosis. To demonstrate this notion, we evaluated the effect of Sp1 overexpression on autophagy and apoptosis induced by the combination treatment. As expected, Sp1 ectopic overexpression in H157 cells attenuated the combination treatment-inducted accumulation of LC3-II and decrease of SQSTM1, and raise of cleaved-caspase-3 and cleaved-PARP (Additional file 3: Figure S7a), also reduced combination treatment-induced the increased of sub G1 population and abated the synergism of curcumin to gefitinib (Additional file 3: Figure S7b and c). Meanwhile, to prove if the induction of autophagy is associated with the downregulation of EGFR, the changes in EGFR localization were confirmed by IF microscopy using the anti-EGFR and anti-LC3 antibodies conjugated to fluorophore. As shown in Fig. 4e, the combination treatment clearly increased LC3 and EGFR co-localization compared to gefitinib or curcumin treatment alone, implying that the two drug combination-induced autophagy can entrap EGFR from plasma membrane into the autophagosome and that trapped proteins may be degraded by lysosome. To further investigate if the combination treatment-induced autophagy may lead to the downregulation of EGFR, we evaluated the levels of EGFR in the presence or absence of autophagy inhibitor (3-MA) and siBeclin-1 treatment. Interestingly, 3-MA and siBeclin-1 treatment restored the decreased activities and the total levels of EGFR induced by gefitinib plus curcumin in H157 cells (Fig. 4f and Additional file 3: Figure S6c), suggesting that there is a crosstalk between EGFR and autophagy. Taken together, these results indicate that gefitinib-induced low level autophagy can be obviously enhanced by combining curcumin and the two drug combination potentiated apoptotic induction, both the biological reactions were medicated through downregulation of Sp1/EGFR activity.

### Curcumin overcomes gefitinib resistance in NSCLC cells through induction of autophagic cell death and autophagy-mediated apoptosis

**Fig. 5:**
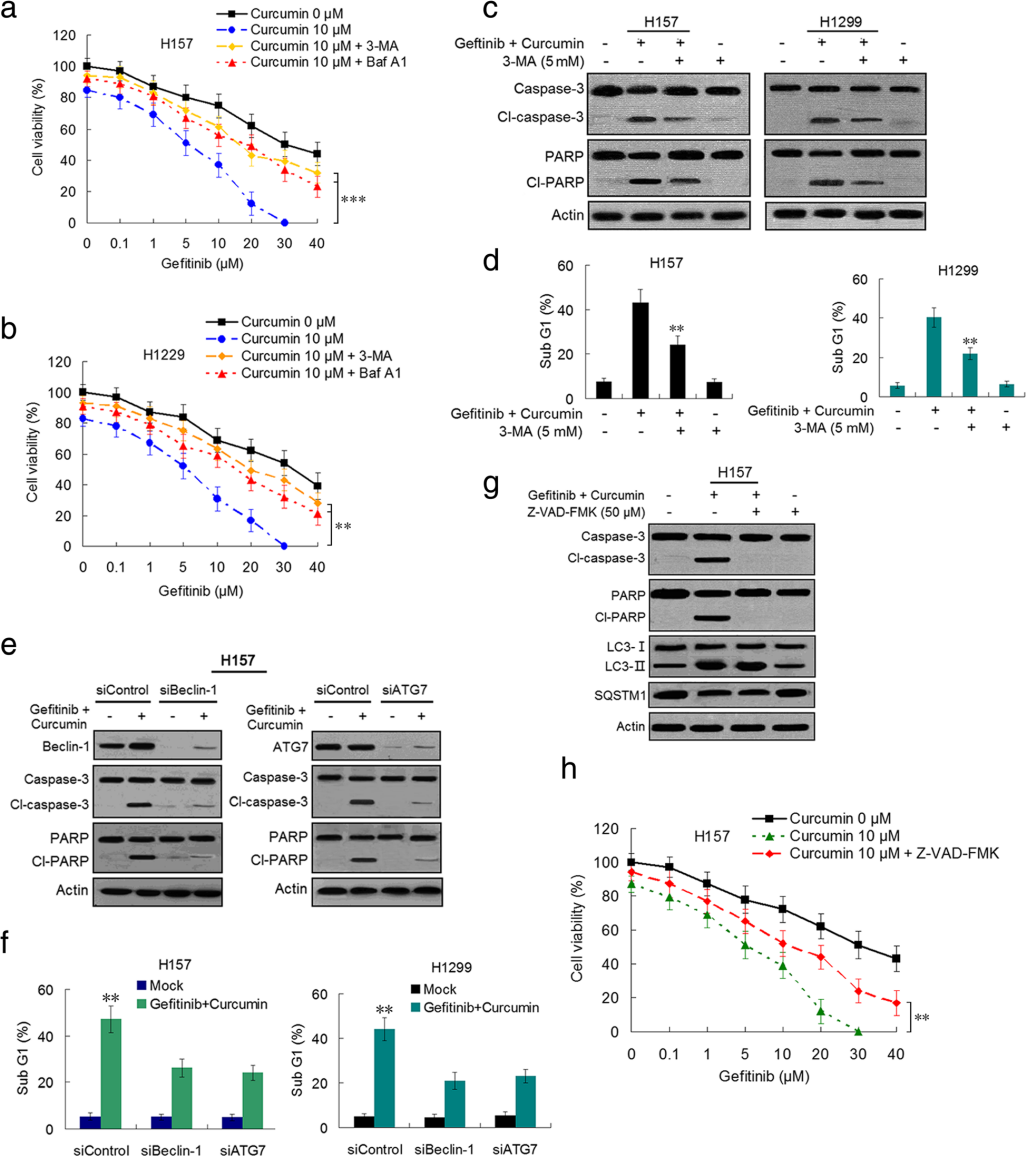
Curcumin reverses gefitinib resistance in NSCLC cells through induction of autophagic cell death and autophagy-mediated apoptosis. **a**-**b** H157 and H1299 cells were treated with gefitinib plus curcumin at indicated concentration for 48 h in the absence or presence of Baf A1 (10 nM) or 3-MA (5 mM), cell viability was detected by CCK-8 assay. **c** H157 and H1299 cells were treated with gefitinib (5 μM) plus curcumin (10 μM) for 48 h in the absence or presence of 3-MA (5 mM). Cleaved (Cl)-caspase-3 and Cl-PARP were determined by immunoblotting. **d** Flow cytometry was used to analyze sub-G1 population in H157 and H1299 cells after drug treatment (5 μM gefitinib plus 10 μM curcumin in combination with or without 5 mM 3-MA). **e** After transfection with siBeclin-1 or siATG7 or siControl, H157 cells were treated with gefitinib (5 μM) plus curcumin (10 μM) for 48 h, Cl-caspase-3 and Cl-PARP were determined by immunoblotting. Similar results were obtained from three independent experiments. Typical immunoblots were presented in the Figure. **f** The sub-G1 population was analyzed by flow cytometry in H157 and H1299 after transfection with siBeclin-1 or siATG7 or siControl . **g** H157 cells were treated with gefitinib (5 μM) plus curcumin (10 μM) in the presence and absence of Z-VAD-MFK, immunoblotting was used to detect apoptosis- and autophagy-related proteins. **h** H157 cells were treated with gefitinib plus curcumin at indicated concentration in the presence or absence of Z-VAD-MFK, cell viability was determined by CCK-8 assay

As shown in the preceding results (Fig. 1c and d), curcumin potently enhanced inhibitory effect of gefitinib on resistant NSCLC cells. Then we showed that the synergism of curcumin to gufitinib in H157 and H1299 cells was reversed by autophagy inhibitor 3-MA or Baf A1 (Fig. 5a and b). Furthermore, curcumin plus gufitinib-induced decreases of colony formation were also returned by 3-MA and Baf A1 in the two cell lines (Additional file 3: Figure S7d). Meantime, gefitinib plus curcumin-induced the increases of cleaved-caspase-3 and cleaved-PARP expressions and caspase-3/7 activity were suppressed by addition of 3-MA (Fig. 5c and Additional file 3: Figure S8a and S9a-b). Moreover, gefitinb plus curcumin markedly increased sub G1 population in the two NSCLC cell lines, which were partly returned by 3-MA (Fig. 5d). In H157 cells, knockdowns of Beclin-1 and ATG7 attenuated gefitinib plus curcumin-induced elevation of cleaved-caspase-3 and cleaved-PARP (Fig. 5e and Additional file 3: Figure S8b). Likewise, increased sub G1 population and enhanced caspase 3/7 activity by gefitinb plus curcumin were also partly returned by silencing Beclin-1 and ATG7 in the two cell lines (Fig. 5f and Additional file 3: Figure S9c-d). In addition, augmented cleaved-caspase-3 and cleaved-PARP by gefitinib plus curcumin were dropped by addition of Z-VAD-FMK, a pan-caspase inhibitor (Fig. 5g and Additional file 3: Figure S8c), also the Z-VAD-FMK partly returned the synergism of curcumin on gefitinib in H157 cells (Fig. 5h). These data indicate that gefitinib plus curcumin-induced apoptosis was autophagy dependent.

### Curcumin enhances therapeutic efficacy of gefitinib and in vivo through Sp1/EGFR downregulation-mediated induction of autophagy-related cell death

**Fig. 6:**
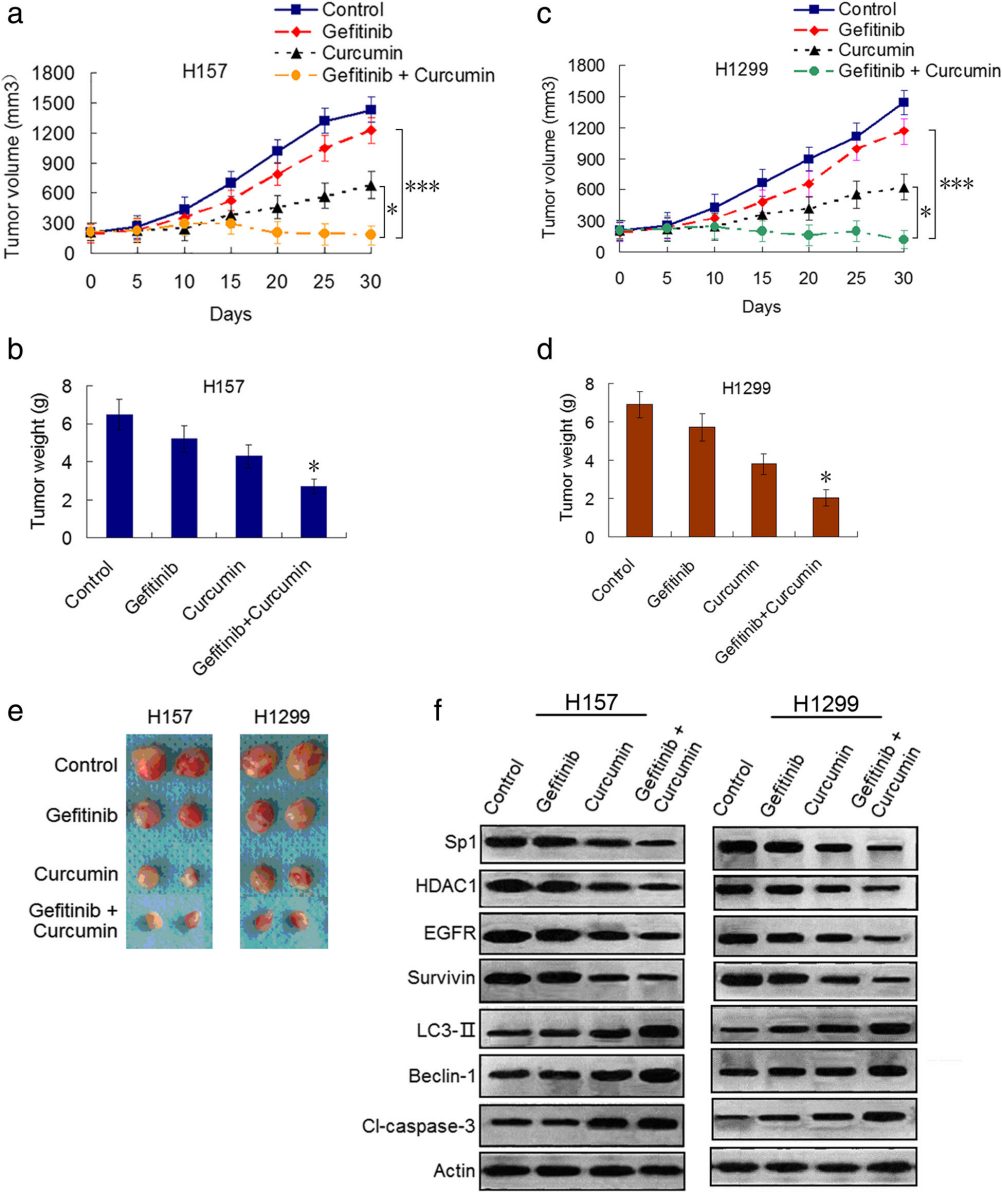
Curcumin enhances therapeutic efficacy of gefitinib in vivo through Sp1/EGFR downregulation-mediated autophagy induction. **a-d** H157 and H1299 cells were subcutaneously injected to the flanks of BALBL/c mice respectively. The mice were treated with vehicle (control), gefitinib, or curcumin, or the two drug combination as described in Materials and Methods. Tumor volume was measured at the time indicated after the onset of treatment. (*P<0.05 and ***P<0.001). The tumor weight was measured at the end of the experiment (*P<0.05 compared with gefitinib, or curcumin treatment alone). **e** Representive pictures of tumor samples from the mice bearing H157 and H1299 cell tumors receiving different treatments as indicated. **f** Immunoblot analysis of the expressions of Sp1, HDAC1, EGFR, survivin, LC3-II, Beclin-1 and cleaved-caspase-3 in H157 and H1299 cell tumor samples from the mice in each treatment group. All experiments were repeated three times. Similar results were obtained from three independent experiments. Typical immunoblots were presented in the Figure

To determine whether curcumin enhanced the efficacy of gefitinib in resistant NSCLC cell tumor in vivo, we established H157 and H1299 NSCLC cell xenograft mouse models as described in Materials and Methods. Tumor-bearing mice were treated with gefitinb and curcumin alone, or combination of two drugs. Gefitinib alone had only minor effect on growth inhibition of the two cell line tumors. Curcumin alone inhibited tumor growth to a greater extent than did treatment with vehicle or gefitinib alone. Combination treatment with the tow drugs markedly inhibited tumor growth compared with that of either single drug treatment (Fig. 6a, c and e). Furthermore, tumor weight was dramatically reduced by treatment with gefitinib in combination with curcumin (Fig. 6 b and d). Finally, we evaluated the effect of gefitinib or curcumin alone, or the two combination on the Sp1/EGFR signaling pathway as well as autophagy and apoptosis associated proteins in vivo. Combination treatment clearly downregutated expressions of Sp1, HDAC1, EGFR and survivin proteins and augmented expressions of LC3-, Beclin-1, and cleavad-caspase-3 compared to single treatment in xenograft specimens (Fig. 6f and Additional file 3: Figure S8d), which were in consistent with the data in vitro. These finding further demonstrated that combination of gefitinib and curcumin can overcome gefitinib resistance in NSCLC through induction of autophagy-related cell death by suppressing Sp1/EGFR signaling.

## Discussion

Although first- and second-generation EGFR-TKIs (including gefitinib, erlotinib and afatinib) produce marked tumor response and improved progression-free survival (PFS) in NSCLC patient harboring activating EGFR mutation [4, 43], patients with wild-type EGFR and KRAS mutation are not sensitive to these EGFR-TKIs [5, 9, 43]. The presence and emergence of drug resistance have highlighted the continued need for combination therapies. In this study, we designed a combinatorial strategy and showed that combination of gefitinib and curcumin exhibited potent inhibitory effect on growth of primary resistant NSCLC cells in vitro and in vivo through downregulating EGFR activity. This process concurrently induced autophagy and apoptosis reactions. We found that synergism of curcumin to gefitinib is more pronounced in gefitinib-resistant cells than in gefitinib-sensitive cells, and gefitinib induced lesser extent downregulation of EGFR activity in gefitinib-resistant cells compared to gefitinib-sensitive cells, suggesting that the insensitivity to gefitinib- induced downregulation of EGFR activity may be involved in gefitinib primary resistance in NSCLC cells, and curcumin reverse gefitinib resistance at least partially through suppressing EGFR activity. Some studies showed that curcumin and its analogs can induce EGFR degradation in EGFR-TKI-resistant and -sensitive lung adenocarcinoma cells and sensitize the TKI-resistant NSCLC cells to gefitinib [33, 47, 48]. Curcumin analog WZ35 was recently reported to induce apoptosis and suppress cell viability via reactive oxygen species (ROS)-mediated activation of the endoplasmic reticulum stress signaling and mitochondrial dysfunction in acquired TKI-resistant NSCLC cells [49]. In human oral squamous cell carcinoma cells, curcumin or its analogs markedly increase gefitinib sensitivity by inducing apoptosis and autophagy [50]. However, the detailed molecular bases for the results were not clarified in these studies. In our model, gefitinib and curcumin combination-induced EGFR downregulation is transcription factors Sp1 and Sp3 dependent. Since Sp1 has been found to regulated EGFR transcription through interacting with HDAC1 in cancer cells [37], we revealed for the first time that combination of gefitinib and curcumin decreased EGFR mRNA level and luciferase activity through suppressing Sp1 and blocking interaction of Sp1 and HDAC1 in the gefitinib-resistant NSCLC cells, and this was accompanied by decreased expression of other Sp1-dependent proteins survivin, VEGF and c-Met, and enhanced induction of autophagy and apoptosis.

EGFR activation trigger the phosphorylation of the inner tyrosine kinase portion of the receptor, then lead to cell proliferation and apoptosis inhibition through activating multiple intracellular signaling pathways, including MAPK/ERS1/2, PI3K/AKT, STAT and SRC/FAK pathways [44]. Also bypass receptor tyrosine kinases, including c-Met, Her2, AXL and IGF1R, play important roles in NSCLC resistance to EGFR-TKIs [7]. It is reported that downregulation of receptor tyrosine kinase by other agents such as HDAC inhibitors can sensitize resistant NSCLC cells to EGFR-TKIs [45, 46]. However, whether curcumin plus gefitinib-induced Sp1/EGFR downregulation affect the receptor tyrosine kinase is not clear. We results showed that curcumin either as a single agent or in combination with gefitinib simultaneously repressed the activity of the receptor tyrosine kinases including their protein and mRNA expressions. In addition, the enhanced activity of EGFR downstream molecules such as ERK1/2, MEK1/2, AKT and S6K was also obviously diminished by the combination treatment. These results suggest that curcumin could reverse NSCLC cell resistance to gefitinib through interfering with multiple bypass receptor tyrosine kinases and the cell proliferation pathways.

Autophagy is a cellular ‘self-eating’ degradation process in which proteins or whole organelles are degraded in lysosomes and recycle to meet the anabolic and bioenergetic need of the cell. In general, autophagy is considered a survival process, in the other condition, autophagy also induces cell death, namely autophagic cell death [11, 51, 52]. Autophagy plays the dual role in response to anticancer treatment, depending on the cellular context and the nature of the treatment. Both EGFR-TKIs and curcumin can induce autophagy [13–16, 21–25]. However, the exact role of autophagy activation induced by EGFR-TKIs remains an area of debate as some studies suggested that blocking autophagy improves the autitcancer activity of EGFR-TKIs to NSCLC cells [13, 14], whereas other studies proposed that induction of autophagy can lead to potentiation of inhibitory effect of EGFR-TKIs on NSCLC cells [16]. In this study, the gefitinib-resistant cells treated with combination of gefitinib and curcumin showed more significant autophagy induction compared to single drug treated cells in parallel with elevation of cleaved-caspase-3 and cleaved-PARP. Importantly, autophagy inhibitors Baf A1 and 3-MA reverse decline of cell viability caused by combining gefitinib with curcumin. Meantime, co-treatment with 3-MA or Baf A1, or knockdown of ATG7 attenuated the two drug combination-induced increase of cleaved-caspase-3 and cleaved-PARP, and partly reduced the two drug combination-induced augmentation of sub G1 population and increase of caspase-3/7 activity in gefitinib-resistant NSCLC cells. Additionally, pan-caspase inhibitor (Z-VAD-FMK) can depress elevated cleaved-caspase-3 and cleaved-PARP induced by curcumin plus gefitinib, and partly returned decrease of cell viability produced by curcumin plus gefitinib, suggesting that the apoptosis induced by the combination treatment depend on autophagy induction. Therefore, we believe that gefitinib and curcumin combination-induced cell death mode consist of autophagic cell death and autophagy-mediated apoptosis. Shen et al. proposed the definition of autophagic cell death. First, cell death occurs without the involvement of the apoptosis machinery. Second, there is an increase of autophagy flux, and not just an increase of the autophagic markers in the dying cells. Finally, pharmacological inhibitors and genetic approaches (ATG knockdown by siRNA transfection) able to rescue or prevent cell death [53]. In this study, combination of gefitinib and curcumin increased autophagy flux. Although autophagy inhibitors (Baf A1 and 3-MA) rescued two drug combination-induced cell death, co-treatment with 3-MA or knockdown of ATG (Beclin-1 and ATG7) partially reversed apoptotic reaction induced by the two drug combination. Moreover, the caspase inhibitor also partly abated increase of cleaved-caspase-3 and cleaved-PARP, and partially rescued cell death induced by the two dug combination. Taken together, these results support the idea that the cell death mode in this study included both autophagic cell death and autophagy-mediated apoptosis.

In addition, we demonstrate that the combination treatment-induced autophagy is mediated via downregulation of Sp1/EGFR as Sp1 ectopic overexpression retarded autophagy and apoptosis. Intriguingly, not only downregulation of EGFR mediated autophagy, but also autophagy induction caused the downregulation of EGFR activity, suggesting that there is an interplay between the autophagy and EGFR in activation process of the two signaling pathways.

## Conclusion

Here, we have provided the first evidence that curcumin and gefitinib synergistically inhibit the growth of gefitinib-resistant NSCLC cells by downregulating Sp1/EGFR activity and the receptor tyrosine kinase pathways, thereby inducing autophagic cell death and autophagy-mediated apoptosis. Our data indicate that curcumin could be used as a sensitizer of EGFR-TKIs in the treatment of NSCLC with wild-type EGFR and KRAS mutation. Further studies are needed to demonstrate the efficacy of the combination treatment of curcumin and EGFR-TKIs in primary EGFR-TKI- resistant NSCLC through clinical trials.

## Additional files

Additional file 1: Supplementary materials and methods. (PDF 100 kb)

Additional file 2: **Table S1.** List of primer sequences used in this study. (PDF 67 kb)

Additional file 3: **Figure S1**. (**a**) H157, (**b**) H1299 and (**c**) PC9 cells were treated with indicated drug combination. The combination index (CI) values for the drugs were calculated according to the Chou-Talalay’s method. (**d**) The knockdowns of Sp1, Sp3, Beclin-1 and ATG7 were verified by immunoblotting. (**e**) The colony formation was determined in H157, H1299 and PC9 cells after treatment with indicated drugs. (* *p* <0.05 and ***p <0.001). **Figure S2.** Quantitative analysis of p-EGFR and EGFR proteins in three NSCLC cell lines after treatment with indicated drugs. **Figure S3.** (**a-c**) The expressions of various proteins indicated in H157 and H1299 cells after treatment with drugs indicated. (**d**) EGFR mRNA expression in H157 and H1299 cells before and after transfection with or without siSp1 or Pcmv6-Sp1. **Figure S4.** Quantitative analysis of proteins indicated in H157 and H1299 cell lines after treatment with indicated drugs. **Figure S5.** Quantitative analysis of proteins indicated in H157 and H1299 cell lines after treatment with different drugs. **Figure S6.** Quantitative analysis of proteins indicated in H157 and H1299 cells after treatment with indicated drugs, or transfection with indicated siRNAs. **Figure S7.** After transfection with control plasmids or pCMV6-Sp1 plasmids, H157 cells were treated with indicated drugs, then (**a**) expressions of proteins indicated, (**b**) sub G1 populations, and (**c**) cell viability were analyzed using the methods described in Materials and Methods. (**d**) The colony formations were determined in H157 and H1299 cells after treatment with indicated drugs (** *P* <0.01 and ***P <0.001). **Figure S8.** Quantitative analysis of proteins indicated in H157 or H1299 cells. **Figure S9.** (**a** and **b**) The caspase-3/7 activities were quantified in H157 and H1299 cells after treatment with indicated drugs, and (**c** and **d**) following transfection with siBeclin-1, or siATG7 or siControl. (PDF 1210 kb)

## Data Availability

All data generated or analysed during this study are included in this published article.

## Abbreviations

3-MA: 3-Methyladenine
AC-H3: Acelylated histone 3
AKT: Protein kinase B
ATG: Autophagy-related gene
Baf A1: Befilomycin A1
Beclin1: Bcl-2-interacting protein1
CCK-8: Cell counting kit-8
CI: Combination index
EGFR: Epidermal growth factor receptor
ERK: Extracellular signal-regulated kinase
H3: Histone 3
HDAC: Histone deacetylase
LC3: microtubule-associated protein 1 light chain 3
MEK: Mitogen-activated extracellular signal-regulated kinase
MIT A: Mithramycin A
NSCLC: Non-small-cell lung cancer
PARP: Poly (ADP-ribose) polymerase
PFS: Progression-free survival
ROS: Reactive oxygen species
siRNA: Small interfering RNA
Sp: Specificity protein
SQSTM1: Sequestosome 1
TKI: Tyrosine kinase inhibitor
VEGF: Vascular endothelial growth factor
