# Supplementary material for: Curcumin overcome primary gefitinib resistance in non-small-cell lung cancer cells through inducing autophagy-related cell death
Source: J Exp Clin Cancer Res. 2019 Jun 13;38:254. doi: 10.1186/s13046-019-1234-8 (PMC6567416; doi:10.1186/s13046-019-1234-8)
Supplement: Supplementary file 1 — Supplementary materials and methods. (PDF 100 kb) [file 13046_2019_1234_MOESM1_ESM.pdf]

Additional file 1:

Supplementary materials and methods

Drugs and antibodies

Gefitinib was from AstraZeneca pharmaceuticals (London, UK). Curcumin, Befilomycin A1 (Baf A1) and 3-Methyladenine (3-MA) were from Sigma-Aldrich (St Louis, MO). Z-VAD-FMK was from Selleck Chemicals (Houston, TX). Antibodies against EGFR, p-EGFR (Y1068), Sp1, Sp2, Sp3, Sp4, VEGF, HDAC1, HDAC2, Acelylated histone 3 (AC-H3) and histone 3 (H3) were from Santa Cruz Biotechnology (Santa Cruz, CA). Antibodies against c-Met, Her-2, AXL, IGF1R, ERK1/2, p-ERK1/2, p-ERK1/2 (T202/Y204), MEK1/2, p-MEK1/2 (S212/218), AKT, p-AKT (S473), S6k, p-S6K (S235/236), microtubule-associated protein 1 Light chain 3 (LC3- Ⅰ), LC3- Ⅱ , SQSTM1, Bclin-1, caspase-3, cleaved-caspase-3, poly (ADP-ribose) Polymerase (PARP), cleaved PARP and Actin were from Abcam (Combride, UK). Second antibodies Alexa Fluor 568 anti-mouse IgG and Alexa Fluor 568 anti-rabbit antibodies were from Jackson ImmunoResearch (Lancaster, PA).

Cell proliferation assay

Proliferation assay were performed using CellTiter 96 Cell Proliferation Assay (MTS) solution following the manufacturer' s protocol (Promega, Madison, WI). Briefly, cells were seeded in 96-well plates at a density of 1000 cells/well. At 24 h, 48 h 72 h and 96 h following addition of drugs, cell proliferation was determined using MTS Reagent according to the manufacturer's protocol. The spectrophotometric absorbance of each sample was measured at 490 nm using the TECAN spectra (Thermo, Austria).

BrdUred incorporation assay

Cell were cultured in RPMI-1640 (10% FBS) medium and after exposure to Gefitinib, bromodeoxyuridine (BrdUred; 10 umo1/L) was added to the medium. One-hour later, the incorporation was analyzed by the BrdUred Incorporation kit (Cell Signaling

Technology, Boston, MA) following the manufacturer's protocol.

Luciferase assays

Cells were transfected with an EGFR promoter luciferase reporter plasmid using Lipofectamine 2000 Transfection Reagent (Invitrogen, Waltham, MA) according to the manufacturer's instructions. After transfection, cells were cultured in 24-well plats in 10% FBS-supplemented RPMI 1640 for 48 h, and subjected to the indicated drugs for 48 h, collected and lysed with passive lysis buffer (Promega, Madison, WI). Aliquots of the lysates (50 μL) were added to media, and luciferase activity was monitored after adding 100 μL of luciferase substrate (Promega, Madison, WI) each well by normalizing to protein concentration and then to control sample transfection with pGL3.

Plasmid transient transfection

The Sp1 and control plasmid DNA (PCMV6XL8) used in the study were obtained from OriGene (Rockville, MD). For transient transfection, $5x10^5$ cells per well were seeded into 6-wells plates and transiently transfected with 2 μg of SP1 plasmids using Lipofectamine 2000 transfection reagent (Invitrogen, Calsbad, CA) following to the manufacturer's instructions. Briefly, plasmid DNA (2 μg) was diluted into 100 μL of RPMI 1640 media. Lipofectamine 2000 reagent (5 μL) was diluted into 100 μL of RPMI 1640 media. The two solutions were then mixed together and incubated at room temperature for 30 min. The total mixed was added to each well (6-well plate) containing 800 μL RPMI 1640 media. Following transfection, cells were allowed to recover for 24 h then stated the treatment.

Immunofluorescence analysis

For the intracellular localization of the proteins, cells were seeded on gelatin-coated coverslips and treated with curcumin, or gefitinib, or curcumin plus gefitinib at the dose as indicated for 24 h. Then cells were fixed with 4% paraformaldehyde for 15 min, permeabilized with 0.1% Triton X-100 for 10 min and blocked with 0.5% BSA

for 30 min. Target proteins in cells were visualized by incubation with the corresponding antibodies overnight at 4, followed by incubation with FITC-conjugated secondary antibodies for 1 h. Rhodamine-conjugated phalloidin and 4', 6-diamidino-2-phenylindole (DAPI) were subsequently used to localized F-actin and nucleus. Images were acquired with a confocal microscope (Leica, Wetzlar, Germany)

For acridine orange staining, cells were treated with the indicated drugs for 48 h, then trypsinized and incubated with acridine orange (AO) at a find concentration of 5 μg/mL for 30min at 37℃, then washed with serum-free media. Image of AO staining were visualized immediately using a confocal microscope.

Caspase activity assays

Caspase activity was determined by using Caspase-Glo 3/7-assay kit (Promega Corporation, Australia). Briefly, cells were seeded in 96-well white luminometer assay plates at a density of $1x10^4$ cells per well and incubated at 37℃. Cells were treated with the indicated drugs for 48 h. 100μL caspase-3/7 reagents were added to each well and incubated for 1 h on rotary shaker at room temperature. The luminescence intensity was measured using a multi-label plate reader (Vector 3; Perkin- Elmer)
