# Supplementary material for: Curcumin overcome primary gefitinib resistance in non-small-cell lung cancer cells through inducing autophagy-related cell death
Source: J Exp Clin Cancer Res. 2019 Jun 13;38:254. doi: 10.1186/s13046-019-1234-8 (PMC6567416; doi:10.1186/s13046-019-1234-8)
Supplement: Supplementary file 2 — Table S1. List of primer sequences used in this study. (PDF 67 kb) [file 13046_2019_1234_MOESM2_ESM.pdf]

# Additional file 2:

**Table S1 List of primer sequences used in this study**

| **Primer for RT-PCR** | | |
|---|---|---|
| **Gene** | **Primer** | **Sequences (5'-3')** |
| Sp1 | forward | ACACGTTCGGATGAGCTACAG |
| | reverse | TGGGCCTCCCTTCTTATTCTG |
| EGFR | forward | ATGCTCTACAACCCCACCA |
| | reverse | GCCCTTCGCACTTCTTACAC |
| c-Met | forward | CCACCCTTTGTTCAGTGTGG |
| | reverse | AGTCAAGGTGCAGCTCTCAT |
| Her2 | forward | GGTGGTCTTTGGGATCCTCA |
| | reverse | ACCTTCACCTTCCTCAGCTC |
| AXL | forward | GGTGGCTGTGAAGACGATGA |
| | | CTCAGATACTCCATGCCA |
| IGF1R | reverse | GACAACCAGAACTTGCAGCA |
| | | CCCTTTAGTCCCCGTCACTT |
| β-actin | forward | GCAAAGACCTGTACGCCAAC |
| | reverse | CTAGAAGCATTTGCGGTGGA |
