# Supplementary material for: Curcumin overcome primary gefitinib resistance in non-small-cell lung cancer cells through inducing autophagy-related cell death
Source: J Exp Clin Cancer Res. 2019 Jun 13;38:254. doi: 10.1186/s13046-019-1234-8 (PMC6567416; doi:10.1186/s13046-019-1234-8)
Supplement: Supplementary file 3 — Figure S1. (a) H157, (b) H1299 and (c) PC9 cells were treated with indicated drug combination. The combination index (CI) values for the drugs were calculated according to the Chou-Talalay’s method. (d) The knockdowns of Sp1, Sp3, Beclin-1 and ATG7 were verified by immunoblotting. (e) The colony formation was determined in H157, H1299 and PC9 cells after treatment with indicated drugs. (* p <0.05 and ***p <0.001). Figure S2. Quantitative analysis of p-EGFR and EGFR proteins in three NSCLC cell lines after treatment with indicated drugs. Figure S3. (a-c) The expressions of various proteins indicated in H157 and H1299 cells after treatment with drugs indicated. (d) EGFR mRNA expression in H157 and H1299 cells before and after transfection with or without siSp1 or Pcmv6-Sp1. Figure S4. Quantitative analysis of proteins indicated in H157 and H1299 cell lines after treatment with indicated drugs. Figure S5. Quantitative analysis of proteins indicated in H157 and H1299 cell lines after treatment with different drugs. Figure S6. Quantitative analysis of proteins indicated in H157 and H1299 cells after treatment with indicated drugs, or transfection with indicated siRNAs. Figure S7. After transfection with control plasmids or pCMV6-Sp1 plasmids, H157 cells were treated with indicated drugs, then (a) expressions of proteins indicated, (b) sub G1 populations, and (c) cell viability were analyzed using the methods described in Materials and Methods. (d) The colony formations were determined in H157 and H1299 cells after treatment with indicated drugs (** P <0.01 and ***P <0.001). Figure S8. Quantitative analysis of proteins indicated in H157 or H1299 cells. Figure S9. (a and b) The caspase-3/7 activities were quantified in H157 and H1299 cells after treatment with indicated drugs, and (c and d) following transfection with siBeclin-1, or siATG7 or siControl. (PDF 1210 kb) [file 13046_2019_1234_MOESM3_ESM.pdf]

Supplementary file 3:

Supplementary Figure S1-9

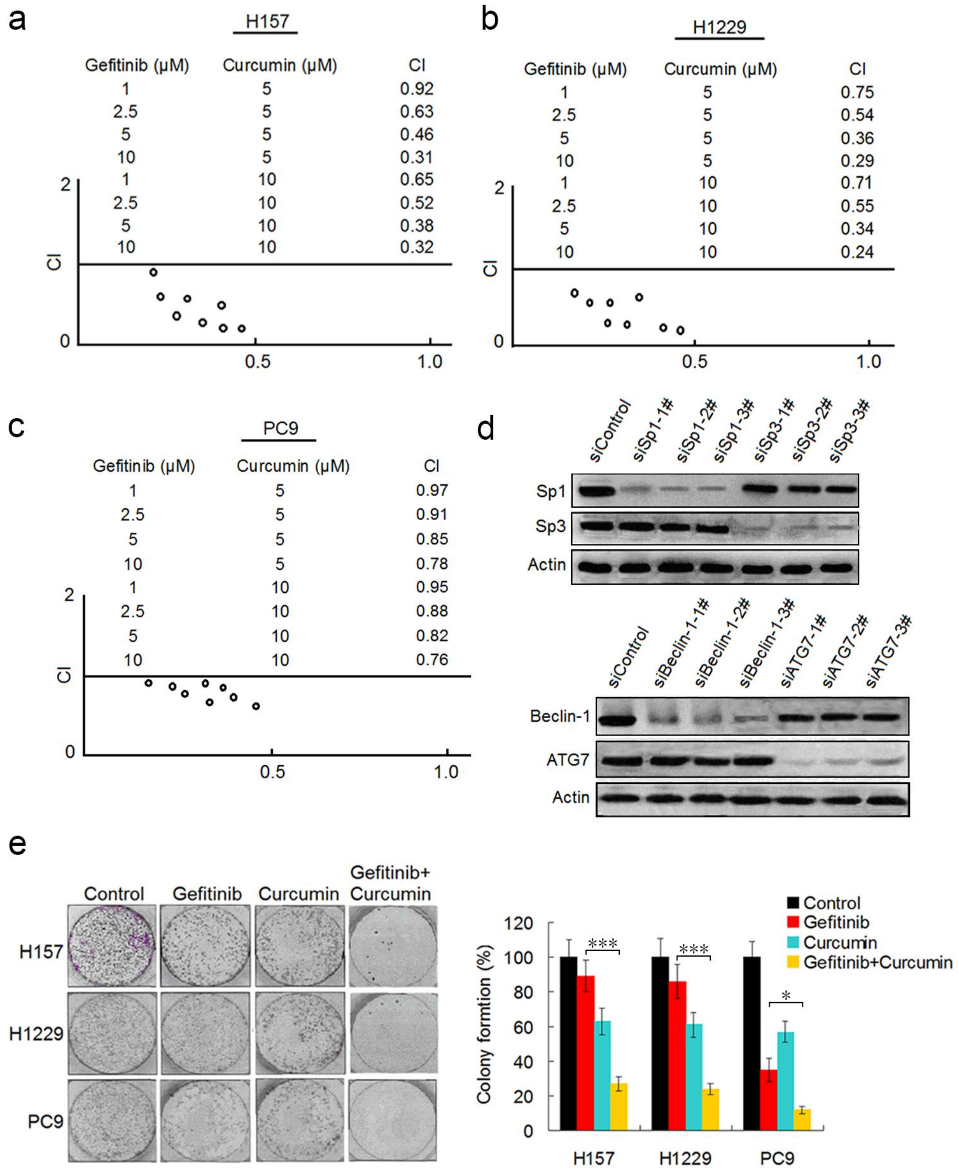

Figure S1

Figure S1 (**a**) H157, (**b**) H1299 and (**c**) PC9 cells were treated with various concentrations of grfitinib and curcumin combination. The combination index (CI) values for gefitinib and curcumin were calculated according to the Chou-Talalay's method at the 48 h time point, with biological response being expressed as the fraction of affected cells. (**d**) Immunoblotting verified the knockdown of Sp1, Sp3,

Beclin-1 and ATG7 by specific siRNAs transfection. (**e**) H157, H1299 and PC9 cells were treated with gefitinib (5 μM), or curcumin (10 μM) alone, or gefitinib plus curcumin for 48 h, then colonogenic assay was performed to determine the colony formation. The percentage of colony formation was calculated by comparing Mock in siControl (designed 100%) (* $p < 0.05$ and *** $p < 0.001$).

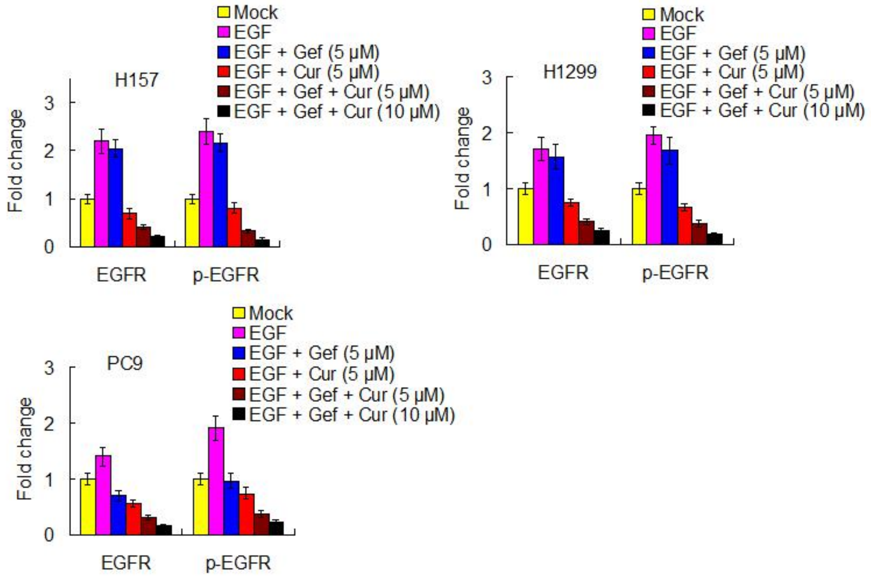

Figure S2

Figure S2 Quantitative analysis of the expression levels of p-EGFR and EGFR in three NSCLC cell lines from immumoblot analysis after treatment with indicated drugs seen in Fig.1f. The fold change of the protein levels compared to control (untreated cells) was determined by a densitometric analysis.

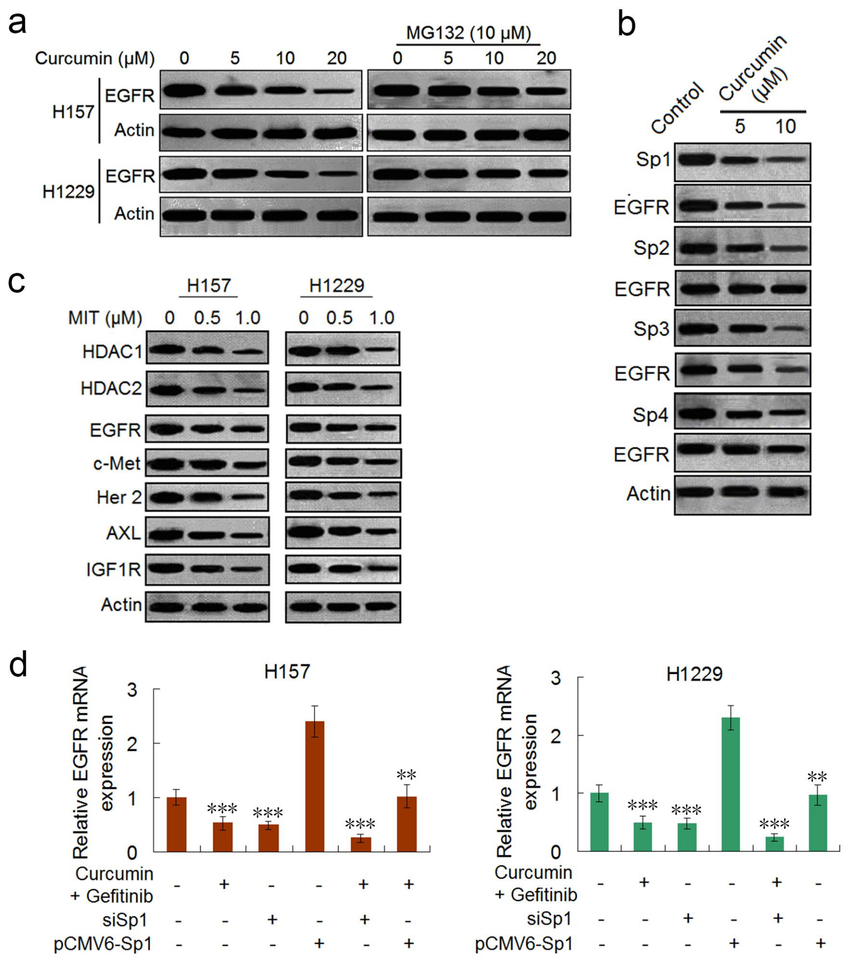

Figure S3

Figure S3 (**a**) H157 and H1299 cells were treated with curcumin at indicated concentration for 48 h in the presence and absence of MG132. The cell lysates were prepared and EGFR protein expressions were analyzed by immunoblotting. (**b**) H157 cells were treated with curcumin at indicated concentration for 48 h. The cell lysates were subjected to immunoblotting to determine the proteins as indicated. (**c**) H157 and H1299 cells were treated with MIT at indicated concentrations for 48 h, and the cell lysates were subjected to immunoblotting to detect HDCA1 and other proteins as indicated. (**d**) Before and after transfection with or without siSp1 or Pcmv6-Sp1, H157 and H1299 cells were treated with gefitinib plus curcumin for 48 h, and mRNA expression was analyzed by real-time quantitative-PCR as described in the Materials and Methods.

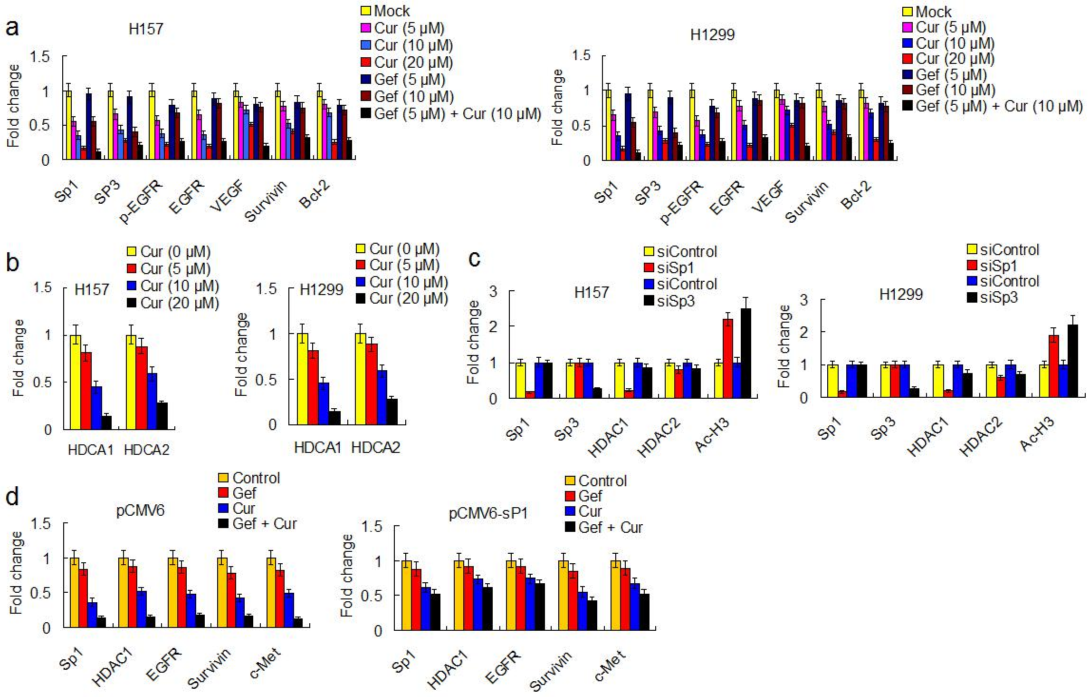

Figure S4

Figure S4 Quantitative analysis of the expression levels of indicated proteins in H157 and H1299 cell lines from immumoblot analysis after treatment with different drugs seen in Fig. 2a (**a**), in Fig. 2b (**b**), in Fig. 2c (**c**), and in Fig. 2d (**d**). The fold change of the protein levels compared to control (untreated cells) was determined by a densitometric analysis.

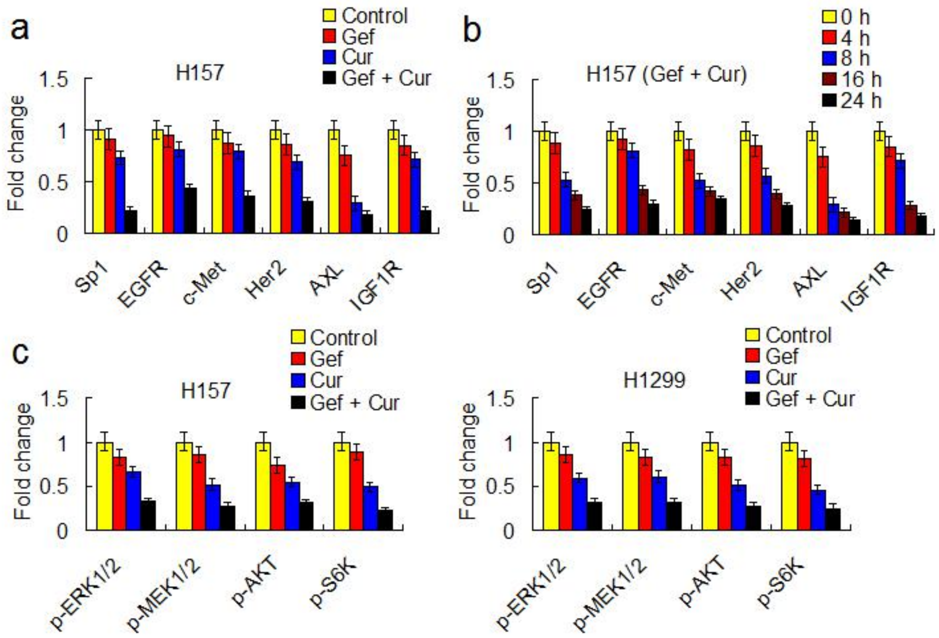

Figure S5

Figure S5 Quantitative analysis of the expression levels of the indicated proteins in H157 and H1299 cell lines from immumoblot analysis after treatment with different drugs seen in Fig. 3a (**a**), in Fig. 3b (**b**), and in Fig. 3c (**c**). The fold change of the protein levels compared to control (untreated cells) was determined by a densitometric analysis. Densitometric values for the phospho-proteins of interest were normalized for protein loading with their total proteins.

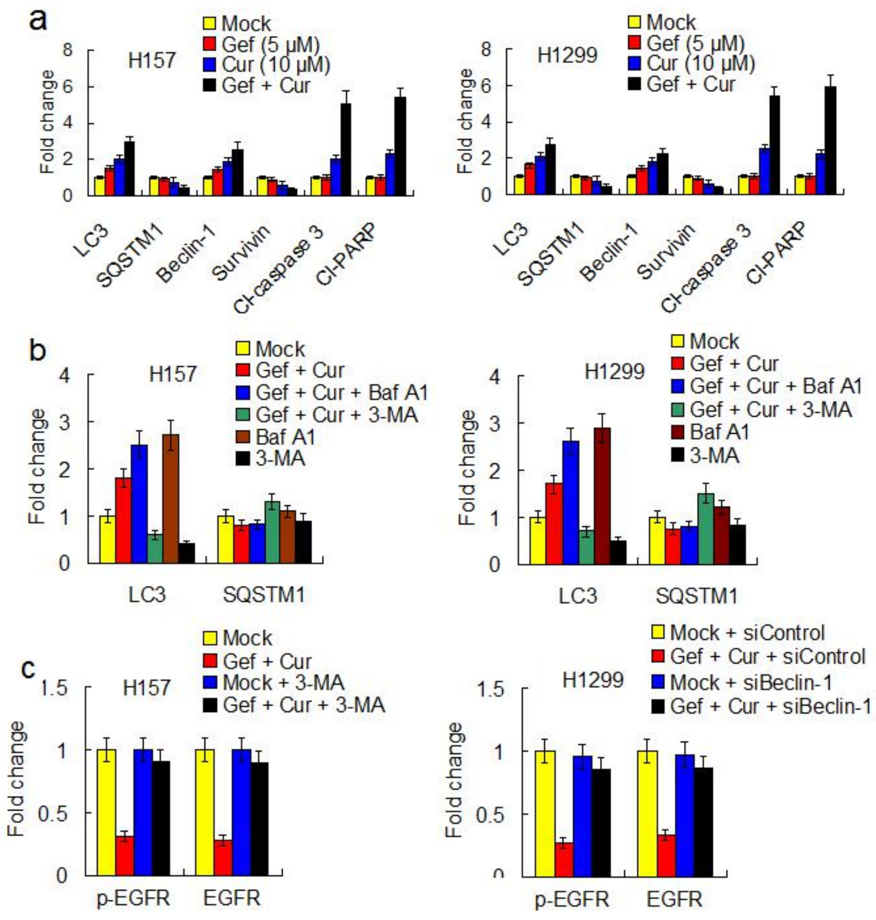

Figure S6

Figure S6 Quantitative analysis of the expression levels of the indicated proteins in H157 and H1299 cell lines from immumoblot analysis after treatment with different drugs, or transfection with indicated siRNAs seen in Fig. 4c (**a**), in Fig. 4d (**b**), and in Fig. 4f (**c**). The fold change of the protein levels compared to control (untreated cells) was determined by a densitometric analysis.

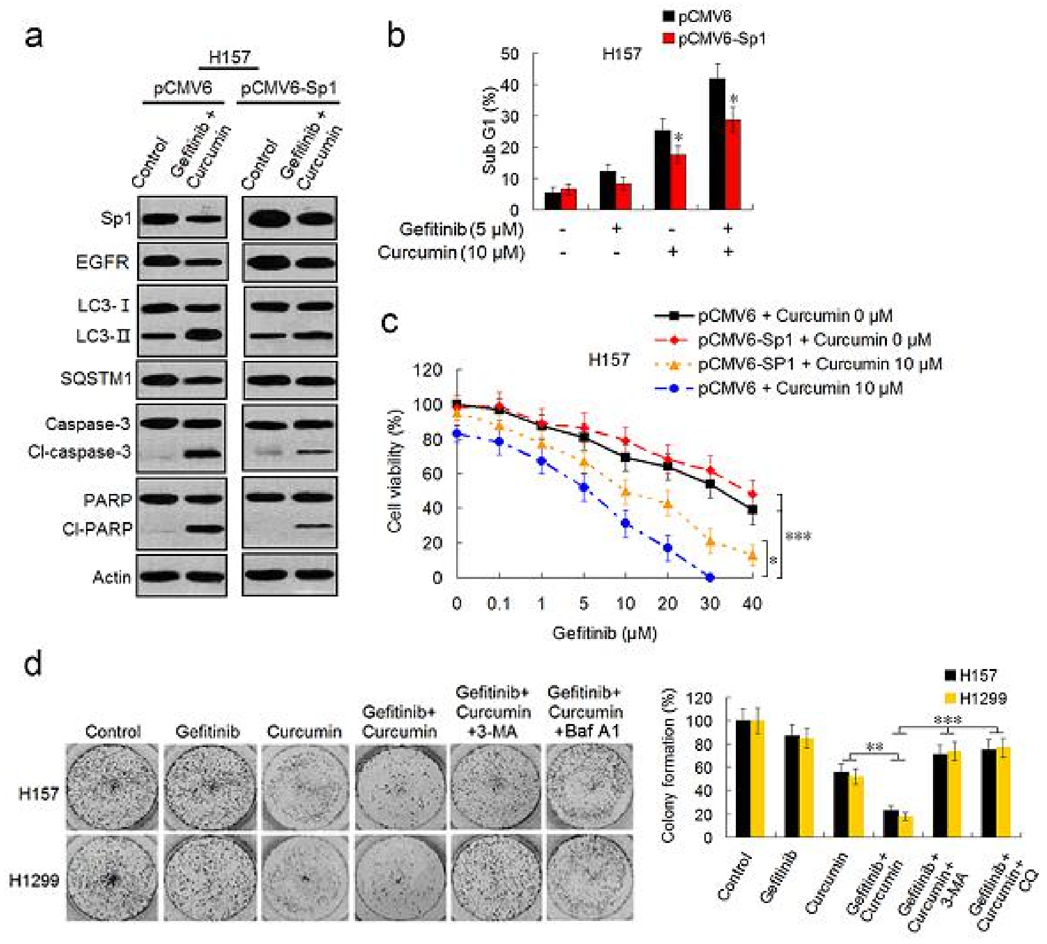

Figure S7

Figure S7 (**a**) H157 cells were transfected with control plasmids (pCMV6) or pCMV6-Sp1 plasmids and treated with gefitinib (5 μM) plus curcumin (10 μM) or Mock for 48 h. Cell lysates were subjected to immunoblotting using the indicated antibodies. (**b**) After transfection with control plasmids or pCMV6-Sp1 plasmids, H157 cells were treated with gefitinib (5 μM) or curcumin (10 μM) alone, or gefitinib plus curcumin for 48 h, and then subjected to Flow cytometry to determine sub G1 population. (**c**) After transfection with control plasmids or pCMV6-Sp1 plasmids, H157 cells were treated with gefitinib, or gefitinib in combination with curcumin at indicated concentration for 48 h, CCK-8 assay was used to detect cell viability. (**d**)

H157 and H1299 cells were treated with gefitinib (5 μM), or curcumin (10 μM) alone, or gefitinib plus curcumin for 48 h in the presence or absence of 3-MA (5 mM) or Baf A1 (10 nM), then clonogenic assay was performed to determine the colony formation. The percentage of colony formation was calculated by comparing Mock in siControl (designed 100%) (** $P < 0.01$ and ***$P < 0.001$).

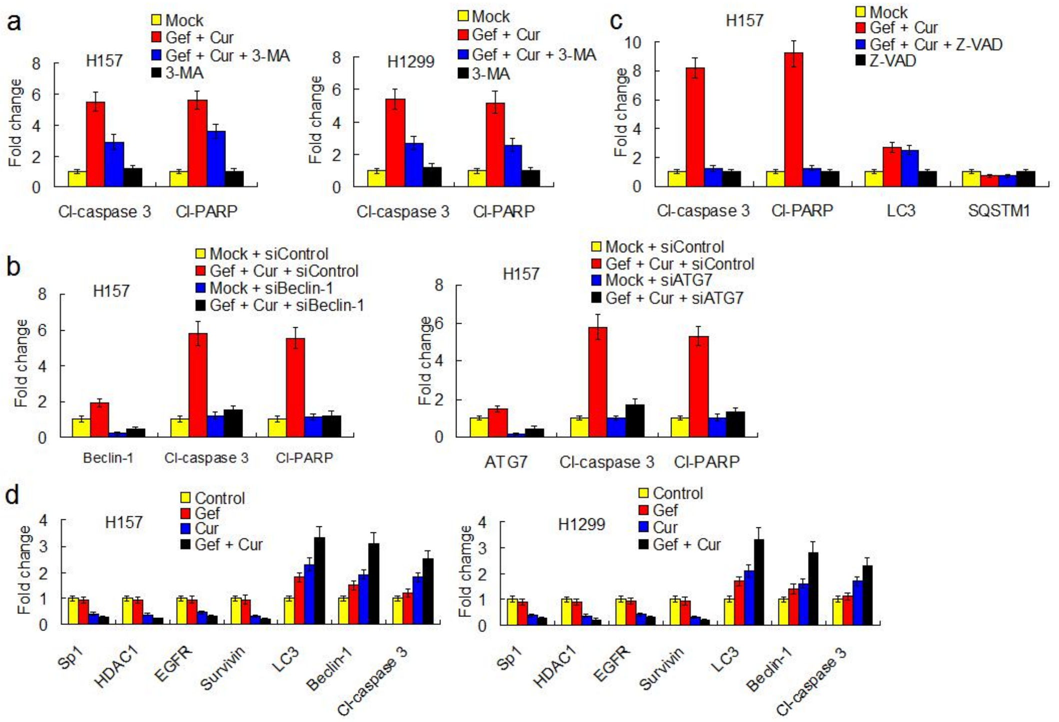

Figure S8

Figure S8 Quantitative analysis of the expression levels of the indicated proteins in H157 or H1299 cell lines from immumoblot analysis after treatment with different drugs, or transfection with indicated siRNAs seen in Fig. 5c (**a**), in Fig. 5e (**b**), in Fig 5g (**c**) and in Fig. 6f (**d**). The fold change of the protein levels compared to control (untreated cells) was determined by a densitometric analysis.

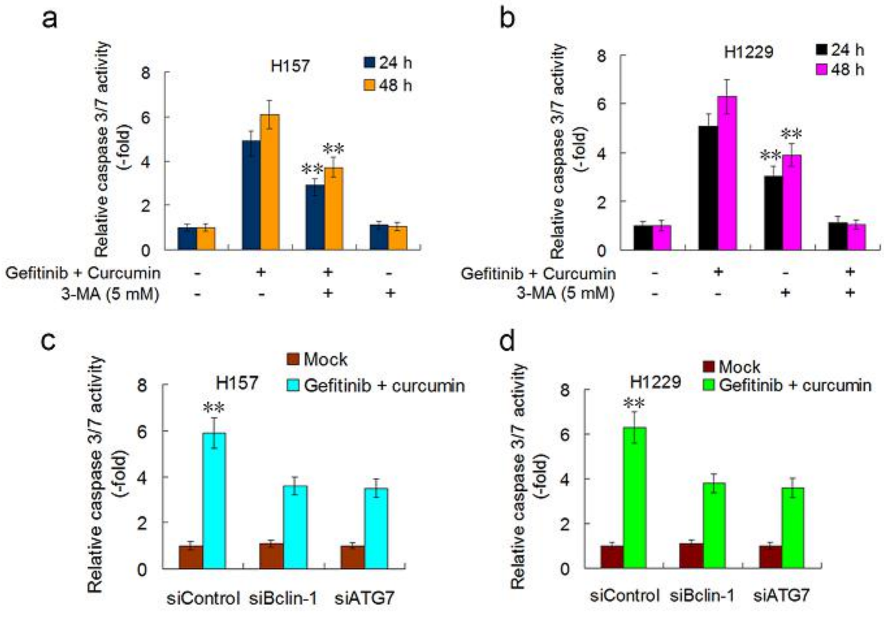

Figure S9

Figure S9 (**a** and **b**) H157 and H1299 cells were treated with gefitinib (5 μM) plus curcumin (10 μM) for 48 h in the presence or absence of 3-MA (5 mM). The caspase-3/7 activities were quantified as described in the Materials and Methods. (**c** and **d**) After transfection with siBeclin-1, or siATG7 or siControl, H157 and H1299 cells were treated with gefitinib (5 μM) plus curcumin (10 μM) for 48 h, The caspase-3/7 activities were quantified as described in the Materials and Methods.
